# Compartmentalization of Telomeres through DNA-scaffolded Phase Separation

**DOI:** 10.1016/j.devcel.2021.12.017

**Published:** 2022-01-24

**Authors:** Amanda Jack, Yoonji Kim, Amy R. Strom, Daniel S.W. Lee, Byron Williams, Jeffrey M. Schaub, Elizabeth H. Kellogg, Ilya J. Finkelstein, Luke S. Ferro, Ahmet Yildiz, Clifford P. Brangwynne

**Affiliations:** 1 Biophysics Graduate Group, University of California, Berkeley CA 94720, USA.; 2 Department of Molecular Biology, Princeton University, Princeton NJ 08544, USA.; 3 Department of Chemical and Biological Engineering, Princeton University, Princeton NJ 08544, USA.; 4 Lewis-Sigler Institute for Integrative Genomics, Princeton University, Princeton NJ 08544, USA.; 5 Molecular Biology and Genetics, Cornell University, Ithaca NY 14850, USA.; 6 Department of Molecular Biosciences and Institute for Cellular and Molecular Biology, The University of Texas at Austin, Austin TX 78712, USA.; 7 Center for Systems and Synthetic Biology, The University of Texas at Austin, Austin TX 78712, USA.; 8 Department of Molecular and Cell Biology, University of California, Berkeley CA 94720, USA.; 9 Physics Department, University of California, Berkeley CA 94720, USA.; 10 Howard Hughes Medical Institute, Chevy Chase MD 20815, USA.

## Abstract

Telomeres form unique nuclear compartments that prevent degradation and fusion of chromosome ends by recruiting shelterin proteins and regulating access of DNA damage repair factors. To understand how these dynamic components protect chromosome ends, we combine *in vivo* biophysical interrogation and *in vitro* reconstitution of human shelterin. We show that shelterin components form multicomponent liquid condensates with selective biomolecular partitioning on telomeric DNA. Tethering and anomalous diffusion prevent multiple telomeres from coalescing into a single condensate in mammalian cells. However, telomeres coalesce when brought into contact via an optogenetic approach. TRF1 and TRF2 subunits of shelterin drive phase separation, and their N-terminal domains specify interactions with telomeric DNA *in vitro*. Telomeric condensates selectively recruit telomere-associated factors and regulate access of DNA damage repair factors. We propose that shelterin mediates phase separation of telomeric chromatin, which underlies the dynamic yet persistent nature of the end-protection mechanism.

## Introduction

The nucleus contains the biological software of the cell – the genome – which is organized into individual chromosomes. Eukaryotic chromosomes end with telomeres, nucleoprotein structures containing repetitive DNA, which protect the genome over successive cell divisions (d'Adda di Fagagna et al., 2003; Maciejowski and de Lange, 2017). Unlike germline cells in which the average telomere length is set, the telomeres in somatic cells shorten over time (Baird et al., 2003; Blackburn, 1991; Harley et al., 1990). This mechanism has been viewed as a tumor-suppressing pathway, as the gradual shortening of telomeres leads to replicative senescence or cell death (Maciejowski and de Lange, 2017).

In humans, telomeres consist of 2-20 kilobases of double-stranded TTAGGG (dsTEL) repeats followed by 50-200 bases of single-stranded telomeric (ssTEL) overhang (Palm and de Lange, 2008). Telomeres associate with the six-protein complex shelterin (Nandakumar and Cech, 2013), which prevents degradation, chromosome end-to-end fusions, and unwanted DNA damage repair (DDR) (d'Adda di Fagagna et al., 2003; Maciejowski and de Lange, 2017). The homologous shelterin components TRF1 and TRF2 specifically bind to dsTEL tracts and recruit other subunits to telomeres. POT1/TPP1 binds to the ssTEL overhang, and TIN2 interconnects TRF1, TRF2, and TPP1. RAP1 binds to the hinge region of TRF2 (Janoušková et al., 2015; O'Connor et al., 2004). These proteins suppress a wide variety of DDR pathways at telomeres by masking the chromosome ends from being improperly recognized as DNA break sites (Galati et al., 2013; Ray et al., 2014). In particular, TRF2 inhibits the ataxia-telangiectasia mutated (ATM) pathway and non-homologous end-joining (NHEJ) of telomeres (Okamoto et al., 2013). Moreover, TRF1 prevents replication fork stalling (Bower and Griffith, 2014; Maestroni et al., 2017), POT1/TPP1 specifically suppresses the ataxia telangiectasia and Rad3-related (ATR) pathway, and TIN2 suppresses ATM, ATR, and NHEJ pathways (Palm and de Lange, 2008).

End-protection by telomeres is mechanistically attributed to the formation of t-loops, wherein TRF2 enables the ssTEL overhang to invade dsTEL tracts and form lasso-like structures (Doksani et al., 2013; Griffith et al., 1999). The t-loop model provides an explanation for how shelterin sequesters the chromosome ends from the ATM and NHEJ pathways (Doksani et al., 2013; Griffith et al., 1999). However, this model does not adequately explain how cells enter senescence while their telomeres still contain kilobases of telomeric repeats (Chiba et al., 2017; Herbig et al., 2004; Smogorzewska et al., 2000) since t-loops have been observed for telomeric DNA as short as one kilobase (Stansel et al., 2001; Kar et al., 2016). Shelterin is also hypothesized to protect telomere ends through the three-dimensional compaction of telomeric chromatin (Bandaria et al., 2016), but decompaction of telomeres upon shelterin knockdown has not been observed by others (Janissen et al., 2018; Timashev et al., 2017). The network of interactions between shelterin components and telomeric DNA could also function as a selectivity barrier to regulate the preferential binding of shelterin and prevention of DNA damage response signaling (Bandaria et al., 2016), but interactions between shelterin and telomeric DNA are too dynamic to serve as a steric barrier. Thus, it remains unclear what physical picture best describes telomere organization and function.

Liquid-liquid phase separation (LLPS) has emerged as a mechanism to create membraneless cellular compartments or “condensates,” such as nucleoli, Cajal bodies, and stress granules (Banani et al., 2017; Shin and Brangwynne, 2017). These structures contain high local concentrations of proteins and nucleic acids that condense into liquid-like assemblies through multivalency and noncovalent interactions, selectively excluding non-interacting molecules (Altmeyer et al., 2015; Riback et al., 2020). LLPS has recently been implicated in controlling chromatin structure (Shin et al., 2018) and in heterochromatin domain formation (Larson et al., 2017; Strom et al., 2017), raising the possibility that telomeric DNA may also form condensates with associated shelterin components. Consistent with this hypothesis, TRF1 and TRF2 display many of the characteristics common in phase separating systems, including intrinsically disordered regions (IDRs), a dimerization domain, and a DNA binding domain (Okamoto et al., 2013; Palm and de Lange, 2008). However, this liquid phase model has not been tested.

Here, we combine intracellular biophysical interrogation and *in vitro* reconstitution to reveal that shelterin components and telomeric DNA organize into liquid-like condensates. Using an optogenetic approach to bring two telomeres together, we find that telomeres are capable of undergoing coalescence, forming a single larger telomeric body. In living cells, we show that telomeres exhibit quantitative signatures of multicomponent LLPS, but their hindered diffusivity results in extremely few coalescence events. We reconstitute the human shelterin complex and find that the interactions between shelterin and telomeric DNA promote the formation of liquid condensates. TRF1 and TRF2 drive phase separation of the shelterin complex, and these liquid droplets selectively recruit telomere-associated factors *in vitro*. We propose that LLPS of shelterin components builds the telomere compartment and could protect chromosome ends by selectively recruiting telomere-associated factors while limiting access of DDR factors.

## Results

### Telomeres in living cells are liquid-like

**Figure 1.**
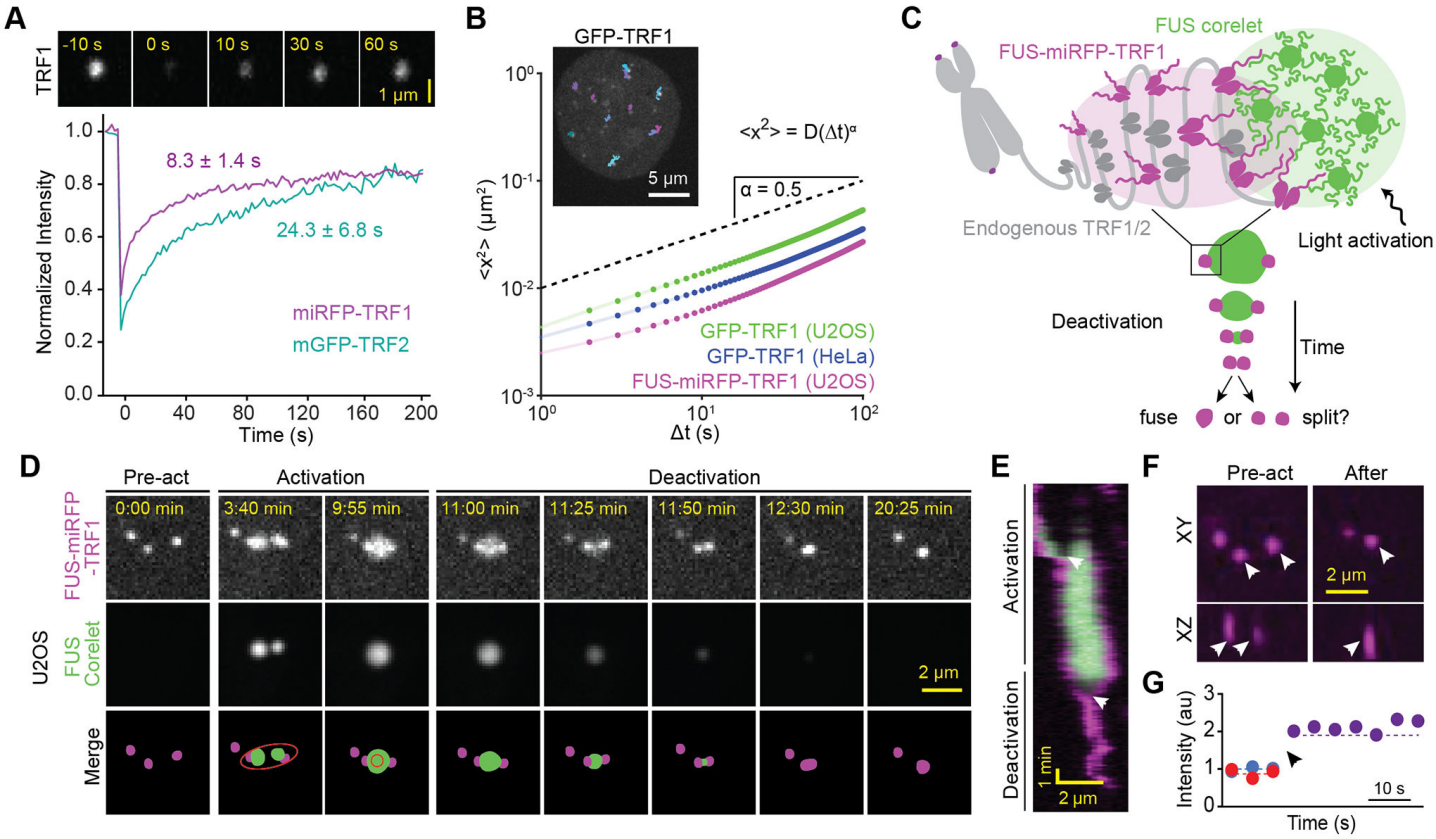
Telomeres in living cells exhibit liquid-like behavior. **A.** (Top) FRAP of miRFP-TRF1 at a telomere in a U2OS cell. (Bottom) Recovery of mGFP-TRF2 or miRFP-TRF1 fluorescence at telomeres in U2OS cells (±SD, n = 9 and 11 telomeres respectively). **B.** (Inset) Trajectories of individual telomeres are colored separately by trajectory duration in a HeLa cell expressing GFP-TRF1. MSD analysis of these trajectories revealed subdiffusive motion with exponent α = 0.54 ± 0.01 and diffusion coefficient D = 2.8 ± 0.1 × 10^−3^ μm^2^ s^−α^ (±SE). The slope of the dashed line serves as a reference for α = 0.5. **C.** Schematic of the optogenetically-induced telomere coalescence experiment: FUS_N_-miRFP-TRF1 serves as a seed at telomeres to recruit FUS_N_ Corelet droplets upon local light activation. After two of these droplets merge, light is deactivated to pull telomeres together as the FUS_N_ droplet shrinks. **D.** Pre-activation, activation, and deactivation of FUS_N_-miRFP-TRF1 and FUS_N_ Corelets in U2OS cells. The ellipse in the schematic merged images shows the local activation pattern. **E.** Kymograph shows that the two telomeres coalesce and remain as a single spot after deactivation. White arrowheads indicate the merging of FUS_N_ Corelet droplets and telomeres. **F.** XY and XZ views of the telomeres before and after activation. White arrowheads mark two telomeres that merge. **G.** The average intensities of the two telomeres add up (dashed lines) as they coalesce (black arrowhead) into a single spot. See also Figure S1 and Movie S1-3.

We first investigated whether telomeres exhibit liquid-like features in human cells. We expressed TRF1 (miRFP-TRF1) and TRF2 (mGFP-TRF2 and miRFP-TRF2) and confirmed that TRF1 and TRF2 formed distinct puncta in nuclei of U2OS cells (Figure S1A-B). As previously reported (Mattern et al., 2004) fluorescence recovery after photobleaching (FRAP) assays showed that TRF1 and TRF2 rapidly exchange between telomeres and the nucleoplasm (Figure 1A), which is typical for phase separating systems (Alshareedah et al., 2021; Taylor et al., 2019). If telomeres are liquid-like, we expect them to coalesce and round up due to surface tension. Consistent with previous studies (Bronshtein et al., 2015; Molenaar et al., 2003; Wang et al., 2008), telomeres exhibit subdiffusive motion and typically do not encounter one another (Figure 1B, Movie S1), likely because they are in a viscoelastic environment and tethered to chromosomes (Feric and Brangwynne, 2013; Lee et al., 2021). Based on mean-squared displacement (MSD) analysis, we estimate that it would take ~5 days for a telomere to reach its nearest neighbor (2.4 ± 1.2 μm, mean ± SD) and as long as ~200 days to reach the average pairwise distance between telomeres (6.8 ± 3.2 μm) via diffusion (Figure S1C-D). Consistently, we were able to detect only one potential coalescence event after imaging 60 cells for 1 h (Figure S1E), demonstrating that telomeres do not frequently merge with one another and remain distinct within living cells due to their suppressed diffusivity.

Due to the infrequency of telomere coalescence, the liquid phase model could not be tested through passive microscopic examination of telomeres in living cells. To controllably pull two or more telomeres into contact, we developed an optogenetic approach based on the Corelet system (Figure 1C) (Bracha et al., 2018). The synthetic Corelet droplets are made by triggering interaction of a phase-separation prone protein (in this case, FUS_N_) with a multivalent (24-mer Ferritin) core through light-triggered heterodimerization between sspB, attached to FUS_N_, and iLID, attached to the core. We tether the droplets to telomeres with FUS_N_-miRFP-TRF1, which binds the telomeric DNA and interacts with the droplet through homotypic FUS_N_ interactions. With light activation, two closely-positioned telomeres can be induced to nucleate FUS_N_ droplets, which fuse to create one FUS_N_ droplet stably interacting with two telomeres (Figure 1D, Movie S2). Following removal of the blue light stimulus (‘deactivation’), the FUS_N_ droplet shrinks, surface tension pulls telomeres inwards, and the telomeres ultimately coalesce into a single spot in three dimensions (Figure 1F). In 46% (11 out of 24) of our attempts, we observed these droplet-guided telomere coalescence events (Figures 1E-G and S1F), which remained a single spot for at least 8 minutes following the dissolution of the FUS_N_ droplets. In several instances, the telomeres detached from the FUS_N_ droplet before contacting each other and relaxed back to their original or more distal positions (Figure S1F-G, Movie S2), indicating that the local viscoelastic constraints on telomeres tend to maintain their relative separation (Shin et al., 2018).

To rule out the possibility that coalescence of telomeres is driven by linking FUS_N_ to TRF1, we linked iLID to TRF1 and FUS_N_ to mCherry-sspB (Figure S1H, Movie S3). In this case, iLID-miRFP-TRF1 only becomes a seed when FUS_N_-mCherry-sspB is bound upon light-activation, and FUS_N_-mCherry-sspB is released from the telomere after deactivation. We observed droplet-guided telomere coalescence events in 10 out of 13 attempts (77%) (Figure S1I-J), demonstrating that observed liquid-like telomere coalescence is driven by the endogenous telomere protein interactions. We observed these merger events in both U2OS (Figure 1D-F) and telomerase-positive hTERT-RPE1 cells (Figure S1F), indicating that telomere coalescence is not due to alternative lengthening of telomeres (ALT)-associated PML bodies (APBs) in U2OS cells (Grobelny et al., 2000; Min et al., 2019; Potts and Yu, 2007; Zhang et al., 2020). Taken together, these data suggest that inducing contact of two telomeres causes their coalescence, which is consistent with telomeres behaving as liquid-like condensates.

### Telomeric DNA acts as an oligomerizing scaffold to promote TRF1 and TRF2-mediated condensation

**Figure 2:**
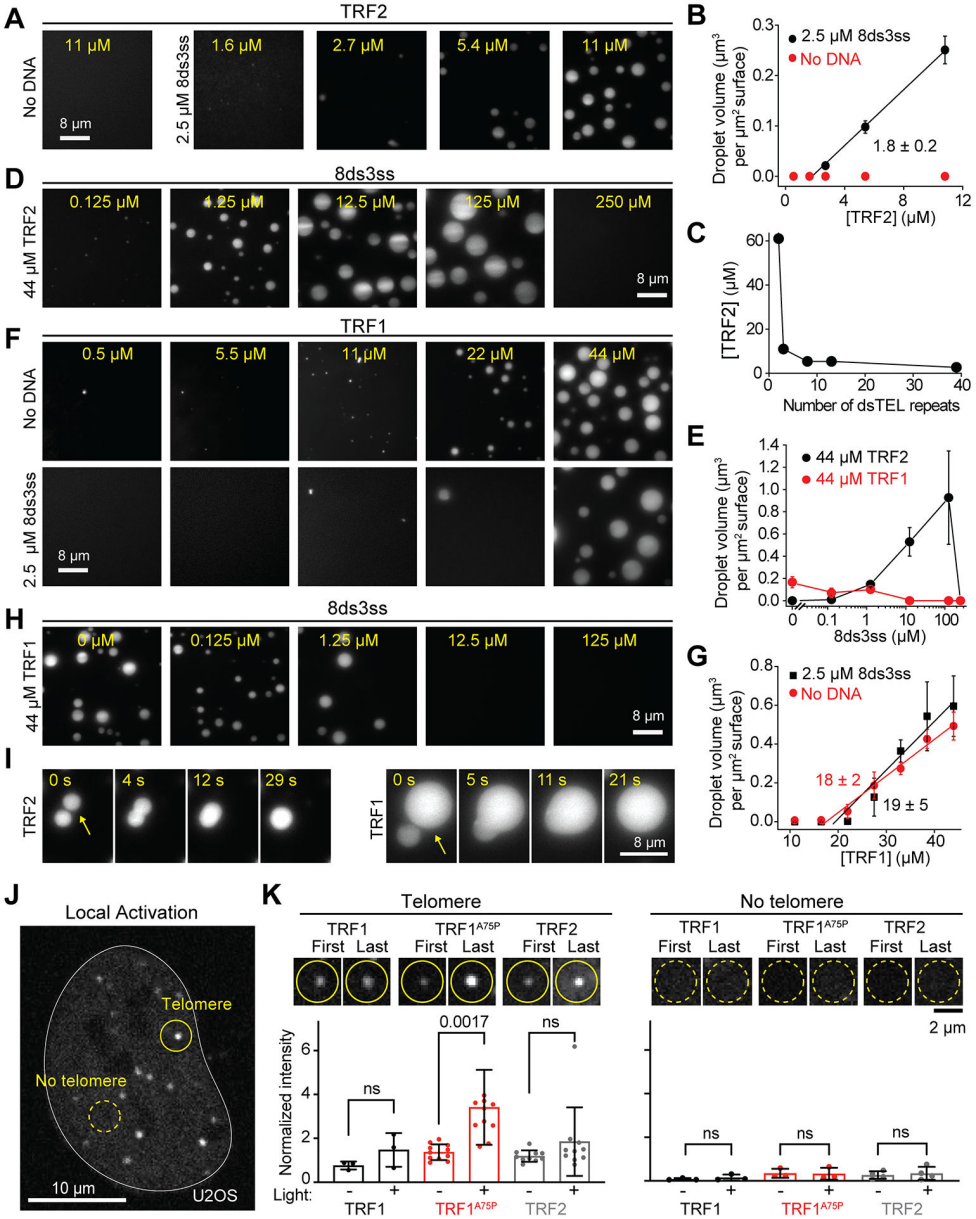
Telomeric DNA acts as an oligomerizing scaffold to promote TRF1 and TRF2-mediated condensation. **A.** Example images of Cy3-TRF2 in the presence and absence of 8ds3ss telomeric DNA. **B.** The total volume of TRF2 condensates settled per micron squared area on the coverslip in the presence or absence of 2.5 μM 8ds3ss (mean ± SD, n = 30 with three technical replicates). Linear fit (solid line) reveals *c*_sat_ (± SE). **C.** The minimum TRF2 concentration that exhibits phase separation with a variable number of dsTEL repeats per DNA substrate. The total concentration of dsTEL tracts was fixed to 20 μM. **D.** TRF2 has a reentrant phase behavior as a function of 8ds3ss concentration. **E.** The total volume of TRF1 or TRF2 condensates settled per micron squared area on the coverslip under variable 8ds3ss concentration (mean ± SD, n = 20 with two technical replicates). **F.** Example images of Cy3-TRF1 in the presence and absence of 8ds3ss. **G.** The total volume of TRF1 condensates settled per micron squared area on the coverslip in the presence or absence of 2.5 μM 8ds3ss (mean ± SD, n = 30 with three technical replicates). Linear fits (solid lines) reveal *c*_sat_ (± SE). **H.** An increase in 8ds3ss concentration inhibits TRF1 phase separation. **I.** Fusion of TRF2 (22 μM, left) and TRF1 (44 μM, right) droplets formed in the presence of 2.5 μM 8ds3ss. **J.** U2OS expressing sspB-mCherry-TRF2. TRF corelets were locally activated at a single telomere (solid circle) or away from any telomere (dotted circle). **K.** (Top) Example images show first and last frames of locally activated TRF1, TRF1^A75P,^ and TRF2 at telomeres (left; n = 3, 11, 10 cells analyzed, respectively) and away from telomeres (right; n = 3, 3, 4 cells analyzed, respectively) in U2OS cells. (Bottom) Quantification of change in intensity upon local activation, at and away from existing telomeres for WT TRF1, TRF1^A75P^, and WT TRF2. The intensity of each locally activated telomere or region was normalized to the average intensity of all other telomeres within the same activated cell. P-values were quantified by one-way ANOVA with multiple comparisons. See also Figures S2 and S3, Table S1, and Movie S4.

To examine whether components of the shelterin complex drive LLPS of telomeres, we purified human shelterin complex proteins and tested if they could create a biomolecular condensate with telomeric DNA *in vitro.* We first characterized whether TRF1 and TRF2 phase separate under physiological salt concentration (150 mM NaCl). While TRF2 did not form liquid droplets in the absence of DNA, the addition of short telomeric DNA with multiple TRF2 binding sites (8 dsTEL and 3 ssTEL repeats; 8ds3ss) initiated the formation of TRF2 droplets (Figures 2A and S2A-C, Table S1). We also observed that TRF2 did not form droplets with nontelomeric DNA at the same length as 8ds3ss, but still formed droplets with a 3-kb long nontelomeric DNA, suggesting that both telomeric DNA sequence and the length of the DNA backbone contribute to TRF2 phase separation.

In the presence of telomeric DNA (2.5 μM 8ds3ss), the volume of the droplets increased linearly with TRF2 concentration (Figures 2B and S2D). The minimum TRF2 concentration that triggered phase separation (*c*_sat_) was 1.8 ± 0.2 μM (Figure 2B, see Methods), which is comparable to the estimated nuclear concentration of TRF2 (~1 μM) and lower than the local concentration of TRF2 at telomeres (>100 μM) (Bandaria et al., 2016; Takai et al., 2010). A telomeric substrate that cannot recruit more than one TRF2 (2ds0ss) nucleated small droplets only at the highest TRF2 concentration tested (61 μM, Figure S2E), whereas increasing the length of the dsTEL tracts to 39 repeats substantially reduced the TRF2 concentration required for triggering phase separation (Figures 2C and S2E, Table S1). These results indicate that the multivalency of the DNA scaffold increases the local concentration of TRF2 dimers and drives phase separation. Interestingly, TRF2 exhibited first an increasing and then decreasing tendency to phase separate as a function of telomeric DNA concentration, and the addition of excess DNA abolished TRF2 condensation (Figure 2D-E). Such reentrant phase behavior has been reported for nucleoprotein assemblies (Banerjee et al., 2017; Sanders et al., 2020; Soranno et al., 2021) and indicates that the stoichiometry of TRF2 and dsTEL repeats is critical for their pairwise interactions to result in phase separation.

TRF1 also formed liquid droplets *in vitro* but under markedly different conditions than TRF2. We found that TRF1 forms liquid droplets in the absence of DNA (Figure 2F). The addition of DNA, either 8ds3ss or nontelomeric DNA, played an inhibitory role in TRF1 phase separation (Figures 2F and S2F), with TRF1 droplets no longer present at increased 8ds3ss concentrations (Figures 2E and 2H). The *c*_sat_ of TRF1 with or without DNA (19 ± 5 μM and 18 ± 2 μM, respectively, Figure 2G) was higher than that of TRF2 with DNA, indicating that TRF1 has a lower propensity to phase separate.

We also found that TRF1 and TRF2 droplets readily dissolve at high salt (0.5 M NaCl, Figure S2G) and coalesce when they encounter each other (Figure 2I), indicating that they exhibit liquid-like material properties. The average fusion time of TRF1 droplets (21 ± 2 s after contact, ±SE) was comparable to that of TRF2 droplets (27 ± 4 s) in the presence of DNA (Figure S2H, Movie S4). TRF1 droplets fused an order of magnitude faster in the absence of DNA (Figure S2H), suggesting that strong interactions between TRF1 with telomeric DNA increase the viscosity of these droplets.

Our *in vitro* findings highlight how telomeric DNA can strongly impact the phase behavior of shelterin components. Because TRF1 phase separates in the absence of DNA (Figure 2F), we used the Corelet system to test whether TRF1-TRF1 interactions could create liquid condensates independent of telomeres in living cells (Figure 2J). We marked telomeres in U2OS cells by expressing miRFP-TRF2, and fused sspB to either TRF1^WT^ or the TRF1 dimerization mutant (TRF1^A75P^) (Bandaria et al., 2016; Fairall et al., 2001) to synthetically oligomerize up to 24 TRF1 molecules on the Ferritin core upon local light activation. When the Ferritin core was recruited to a single telomere, enrichment of TRF1^WT^ or TRF1^A75P^ at that telomere was slightly increased (Figure 2K). Interestingly, *de novo* TRF1 puncta were not observed to form when we locally activated a region away from a telomere (Figure 2K), except under very high expression conditions (Figure S2I-J). This is consistent with the concept that high concentration and valency are required for shelterin-mediated phase separation. We also obtained similar results with TRF2 in both U2OS and hTERT-RPE1 cell lines (Figures 2K and S2I-J), demonstrating that TRF1 and TRF2 condensation is dependent on multivalent interactions with the telomeric DNA in living cells.

### Differences in TRF1 and TRF2 phase separation are driven by their N-terminal domains.

**Figure 3.**
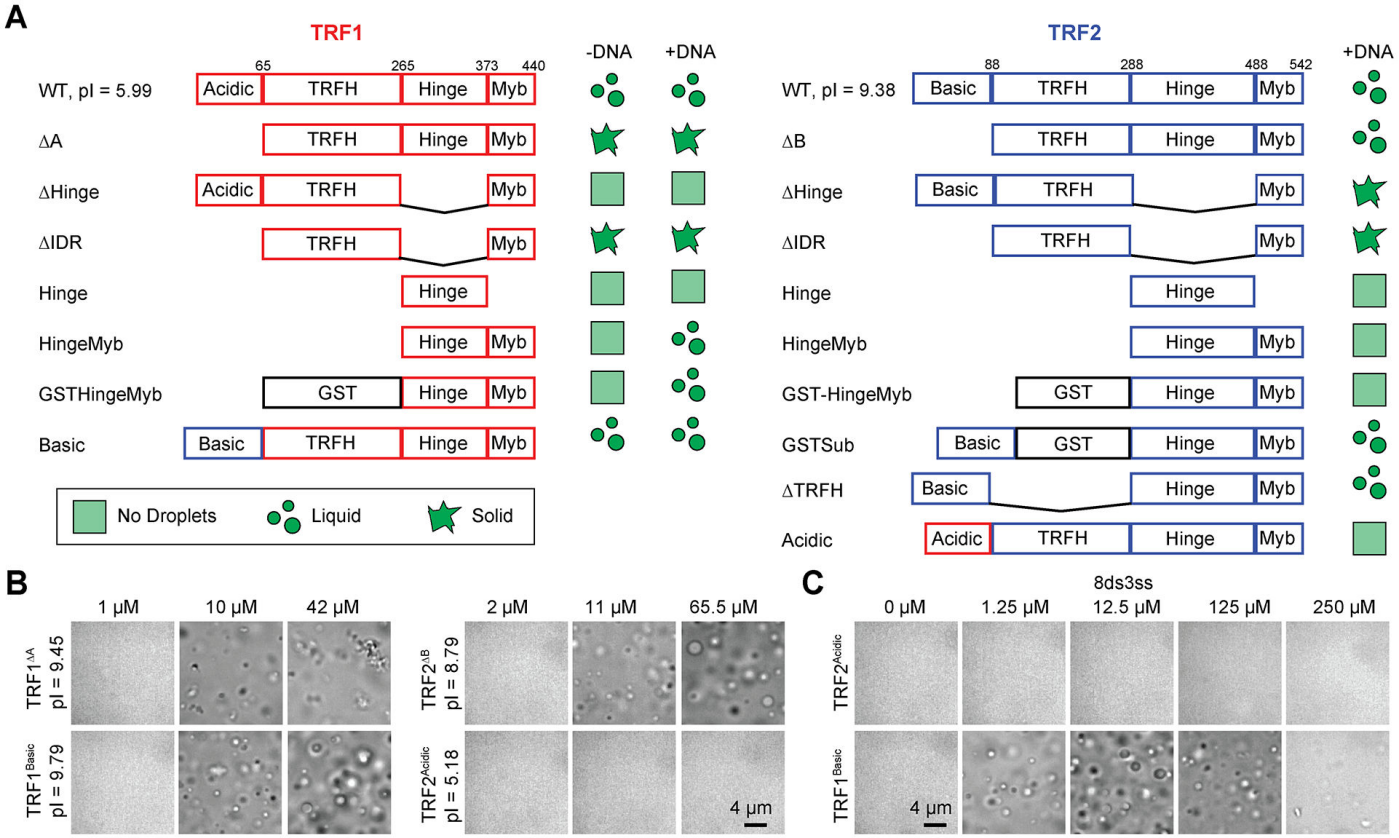
Differential drivers of TRF1 and TRF2 phase separation. **A.** Domain organization and condensate state of full-length, truncated, and engineered TRF1 and TRF2 constructs in the presence and absence of 2.5 μM 8ds3ss (pI: isoelectric point). **B.** Brightfield images taken in the presence of 2.5 μM 8ds3ss DNA show that TRF1^ΔA^, TRF1^Basic^, and TRF2^ΔB^ form condensates, whereas TRF2^Acidic^ does not form condensates. **C.** TRF1^Basic^ phase separation exhibits reentrant response to 0 - 250 μM 8ds3ss, whereas TRF2^Acidic^ does not phase separate in any conditions (protein concentration was set to 20.1 μM). See also Figure S3.

TRF1 and TRF2 are homologous proteins with flexible N-terminal charged domains (acidic in TRF1; basic in TRF2), structured TRFH dimerization domains (Court et al., 2005), flexible hinge domains, and C-terminal DNA-binding Myb domains (Figure 3A) (de Lange, 2018). To investigate which domains were primarily responsible for phase separation, we generated fragments and domain swapping mutants of TRF1 and TRF2 (Figures 3A and S3A-B). Deleting the N-terminal acidic domain of TRF1 (TRF1^ΔA^) triggered the formation of irregularly shaped, solid-like condensates across conditions, underscoring its role in the solubility of TRF1 (Figure 3B). Interestingly, replacing the acidic domain of TRF1 with the basic domain of TRF2 (TRF1^Basic^) resulted in a reentrant response of phase separation to DNA concentration, similar to TRF2^WT^ (Figure 3C). In addition, swapping the acidic domain of TRF1 into TRF2 (TRF2^Acidic^) inhibited phase separation of TRF2 in the presence of DNA (Figures 3B-C and S3D), similar to TRF1^WT^. These results suggest that the TRF2 basic domain promotes phase separation through its interactions with telomeric DNA (Biffi et al., 2012; Poulet et al., 2009), whereas the TRF1 acidic domain weakens electrostatic interactions with the DNA backbone and reduces phase separation in the presence of DNA. We also observed that removing the basic domain from TRF2 (TRF2^ΔB^) did not strongly change its phase behavior (Figure 3B), presumably because this construct is still highly positively charged and the TRFH domain of TRF2 may be sufficient for favorable interactions with telomeric DNA (Amiard et al., 2007) in the absence of the basic domain. Differences in phase separation were not due to reduced DNA binding, as these mutants maintained a high affinity to bind telomeric DNA (Figure S3B).

We also tested the possible roles of IDRs and dimerization domains in TRF1 and TRF2 phase separation. Deletion of the hinge (ΔHinge) or both the hinge and N-terminal domains (ΔIDR) reduced the solubility and inhibited LLPS of TRF1 and TRF2 (Figures S3C and E). While Hinge-Myb of TRF2 was unable to drive LLPS in the presence or absence of DNA, Hinge-Myb of TRF1 formed droplets only at very high concentrations (>100 μM) (Figure S3C). Artificial dimerization of Hinge-Myb triggered phase separation in TRF1 with telomeric DNA, but additionally required the N-terminal basic domain in TRF2 (GSTSub) for phase separation (Figures 3A, S3C and F). Taken together, dimerization and IDRs of TRF1 and TRF2 are essential for phase separation, and the N-terminal domain regulates interactions with telomeric DNA in liquid droplets.

### The shelterin complex phase separates *in vitro*

**Figure 4.**
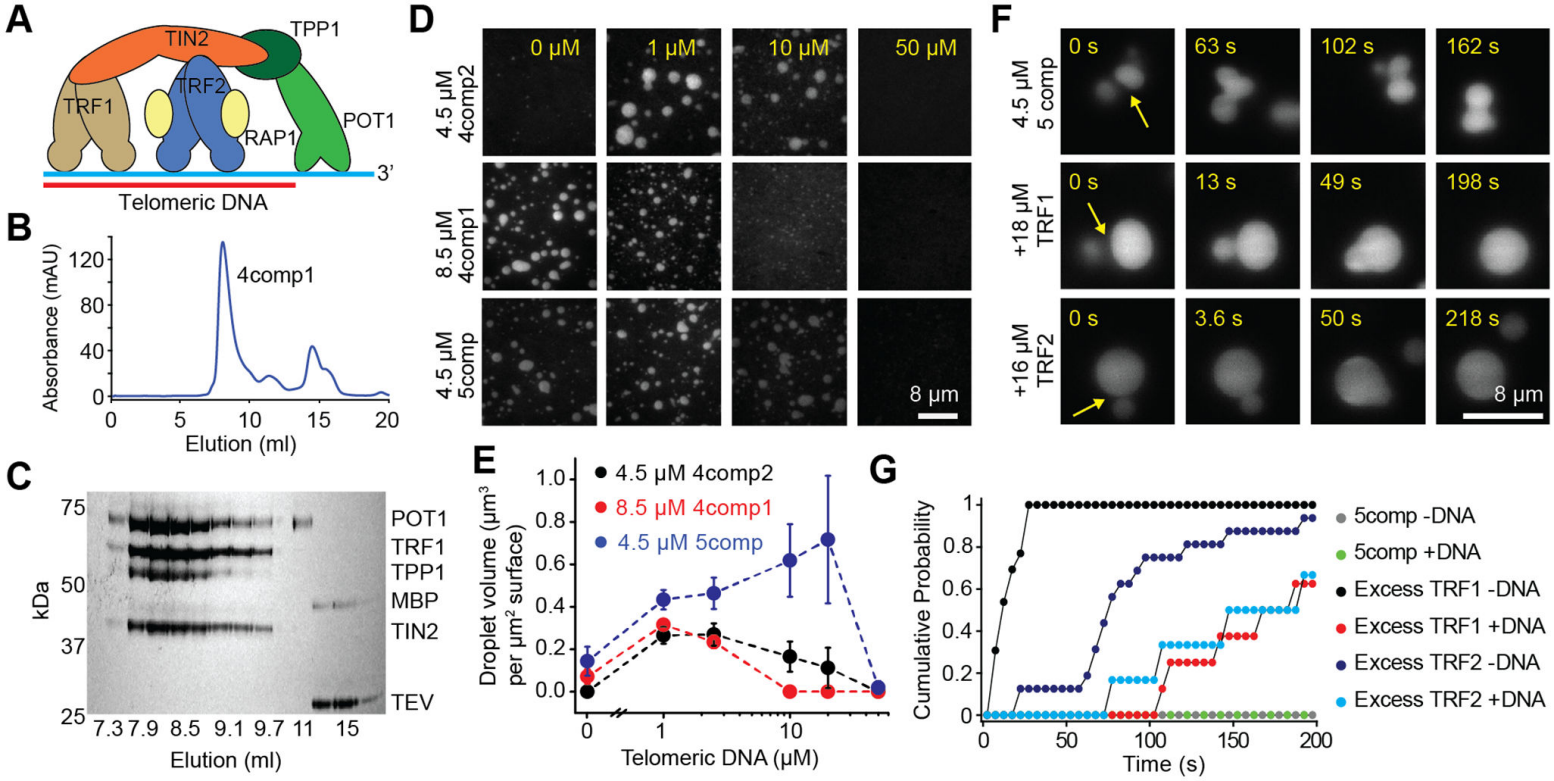
The shelterin complex phase separates *in vitro*. **A.** A schematic of the human shelterin complex. TRF1 and TRF2 are homodimers that bind to dsTEL, and POT1/TPP1 binds to ssTEL. TIN2 interconnects TRF1, TRF2, and TPP1, and RAP1 binds to TRF2. **B-C.** UV absorbance (B) and denaturing gel (C) show that 4comp1 elutes as a single complex from a gel filtration column. **D.** 4comp2 and 5comp exhibit reentrant responses to increasing DNA concentration similar to TRF2 droplets, while 4comp1 is inhibited by increasing DNA concentration similar to TRF1 droplets. **E.** The total volume of shelterin droplets settled per micron squared area on the coverslip under variable 8ds3ss concentration (mean ± SD, n = 20 with two technical replicates). **F.** In the presence of 2.5 μM 8ds3ss, 5comp droplets do not fuse on relevant time scales, whereas the addition of excess TRF1 or TRF2 reduces the fusion time. **G.** Cumulative probability of 5comp droplet fusion in the presence and absence of excess TRF1 or TRF2 after forming a contact at t = 0 s (n = 7, 4, 13, 16, 15 and 7 events from top to bottom). See also Figure S4 and Movie S4.

To examine the role of interactions among TRF1, TRF2, and the rest of the shelterin components in driving telomeric phase separation, we co-expressed human shelterin components in insect cells (Figure 4A). Four component complexes containing TRF1, TIN2, TPP1, and POT1 (4comp1); TRF2, TIN2, TPP1, and POT1 (4comp2); and the five-component complex that contains both TRF1 and TRF2 (5comp) eluted as a stable complex from gel filtration (Figures 4B-C and S4A). Unlike TRF1 and TRF2, neither POT1 nor co-purified TPP1 and TIN2 formed liquid condensates (Figure S4C), showing that these proteins do not phase separate on their own. Although POT1 does not specifically bind to TRF1 or TRF2 (Lim et al., 2017; Liu et al., 2004), it partitioned into TRF1 and TRF2 droplets (Figure S4D-E), indicating that interactions within the condensate can recruit POT1 in the absence of TPP1-TIN2 and ssTEL tracts.

We then characterized the phase behavior of the shelterin complexes with and without telomeric DNA (Figures 4D and S4F). Similar to TRF1 condensates, 4comp1 efficiently formed liquid droplets in the absence of telomeric DNA, and its phase separation was inhibited by the addition of telomeric DNA. Similar to TRF2 condensates, 4comp2 only phase-separated in the presence of DNA and formed droplets across a range of telomeric DNA concentrations (Figures 4D-E and S4F). 5comp, containing equimolar TRF1 and TRF2, phase-separated across a broader range of telomeric DNA (8ds3ss) concentrations (Figure 4D) and in the absence of DNA (Figure S4F). Therefore, TRF1 and TRF2 are synergistic in driving phase separation of shelterin, possibly due to complex coacervation between their charged domains.

Next, we investigated the material state of the shelterin condensates *in vitro*. 5comp droplets could adhere to one another and change shape, but they could not complete fusion into a spherical droplet within 200 s (Figure 4F, Movie S4). Therefore, shelterin droplets appear to be more viscoelastic than TRF1 or TRF2 droplets and exhibit gel-like properties in the presence of telomeric DNA. Because shelterin components form subcomplexes with fewer subunits (de Lange, 2018), and TRF1 and TRF2 are more abundant than POT1-TPP1 at telomeres (Takai et al., 2010), we asked whether changing the stoichiometry of shelterin subunits affect these condensates. The equimolar mixtures of separately purified TRF1, TRF2, TPP1-TIN2, and POT1 also formed liquid droplets with all components present and responded to changes in DNA concentration similar to co-purified 5comp (Figure S4G-H). The addition of ~3-fold excess TRF1 or TRF2 substantially reduced fusion times of shelterin droplets (Figure 4F-G and S4I-K). These results suggest that the stoichiometry of shelterin components could serve to regulate the viscoelasticity of telomeres *in vivo*.

### Telomeres exhibit quantitative signatures of multicomponent liquids

**Figure 5.**
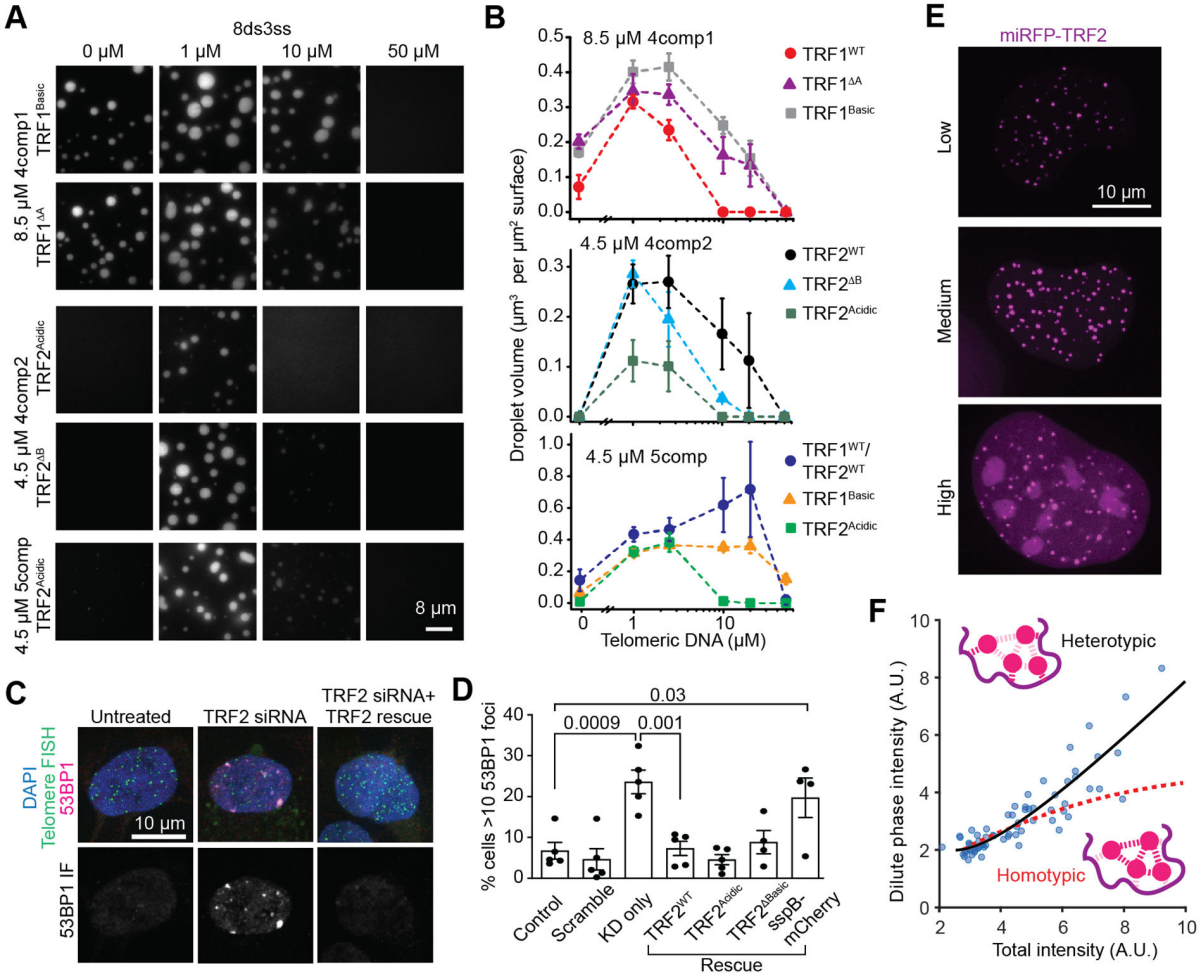
Telomeric condensates exhibit quantitative signatures consistent with multicomponent phase-separated liquids both *in vitro* and in living cells. **A.** Example images show phase separation of 4comp1, 4comp2, and 5comp assembled using N-terminal swap or truncation mutants of TRF1 and TRF2. **B.** The total volume of shelterin droplets assembled with native or mutant TRF1 and TRF2 settled per micron squared area on the coverslip under different 8ds3ss concentrations (mean ± SD, n = 20 with two technical replicates). **C.** 53BP1 staining (magenta) of hTERT-RPE1 cells that are treated or untreated with TRF2 siRNA. Telomeres are stained with a telomeric DNA FISH probe (green). Nuclei are labeled with DAPI (blue). **D.** The percentage of hTERT-RPE1 cells with more than 10 53BP1 foci per nucleus under knockdown and rescue conditions. Error bars represent SEM of five biological replicates for all conditions except for TRF2^ΔB^ and sspB-mCherry (four replicates). n > 1000 cells analyzed for all conditions. P-values were calculated by one-way ANOVA with multiple comparisons. **E.** Overexpression of miRFP-TRF2 leads to an increased dilute phase (nucleoplasmic) partitioning in U2OS cells. **F.** The dilute phase intensity increases nonlinearly as a function of the total intensity of the miRFP-TRF2 signal in U2OS cells. The data fit to a nonlinear heterotypically stabilized model (black solid curve) but not to homotypic interactions (red dashed curve). The ‘homotypic’ curve is not a flat line due to the presence of endogenous protein (see Riback et al., 2020). See also Figure S5.

To determine how altered phase behavior of TRF1 and TRF2 mutants might affect phase separation of shelterin complexes *in vitro*, we assembled shelterin complexes using N-terminal swap or deletion mutants of TRF1 and TRF2 (Figure S5A). Replacing TRF1^WT^ with TRF1^ΔA^ or TRF1^Basic^ resulted in phase separation of 4comp1 over a wider range of DNA concentrations (Figure 5A-B). Replacing TRF2^WT^ with TRF2^ΔB^ in 4comp2 and 5comp did not inhibit phase separation (Figure 5A-B and S5B), but adding TRF2^Acidic^ reduced the size and number of droplets of the complex (Figures 5A-B).

We next tested whether altered phase separation of TRF2 affects end-protection of telomeres in living cells, by expressing TRF2^WT^, TRF2^ΔB^, or TRF2^Acidic^ upon knockdown of endogenous TRF2 in hTERT-RPE1 cells (Figure 5C-D, Figure S5C-F). We quantified the number of DNA damage foci formed by the localization of p53 binding protein 1 (53BP1), a downstream signaling protein recruited to these DDR foci (Figure 5C) (Schultz et al., 2000). As previously reported (Takai et al., 2003), knockdown of endogenous TRF2 led to a significant increase in the percent of nuclei with greater than ten 53BP1 foci (23.6% compared to 6.7% of untreated cells, Figures 5D and S5C-F). Consistent with previous studies (Okamoto et al., 2013; Rai et al., 2016), both TRF2^WT^ and TRF2^ΔB^ rescued telomere end-protection, with few cells exhibiting greater than ten 53BP1 foci (7.3% and 8.8%, respectively; Figure 5D). We found that TRF2^Acidic^ also rescued end-protection (4.5%, Figure 5D), consistent with phase separation of this mutant with the rest of the shelterin complex *in vitro*.

To further probe whether telomere compartmentalization requires interactions between multiple components in living cells, we quantified the relative importance of homotypic vs heterotypic interactions in telomere formation in U2OS cells. We measured the nucleoplasmic, or dilute phase concentration (*c*_dil_) of miRFP-TRF2 at increasing expression levels (Figure 5E-F). If homotypic interactions of miRFP-TRF2 dominate its phase separation, *c*_dil_ would remain constant as miRFP-TRF2 concentration is increased (Riback et al., 2020). However, we observed that *c*_dil_ continues to increase with miRFP-TRF2 overexpression (Figure 5E-F). Our results suggest that telomeres are thermodynamically stabilized by heterotypic interactions, which is consistent with the necessity of telomeric DNA for shelterin condensation in live cells.

### Phase separation of shelterin is modulated by telomere-associated factors

**Figure 6.**
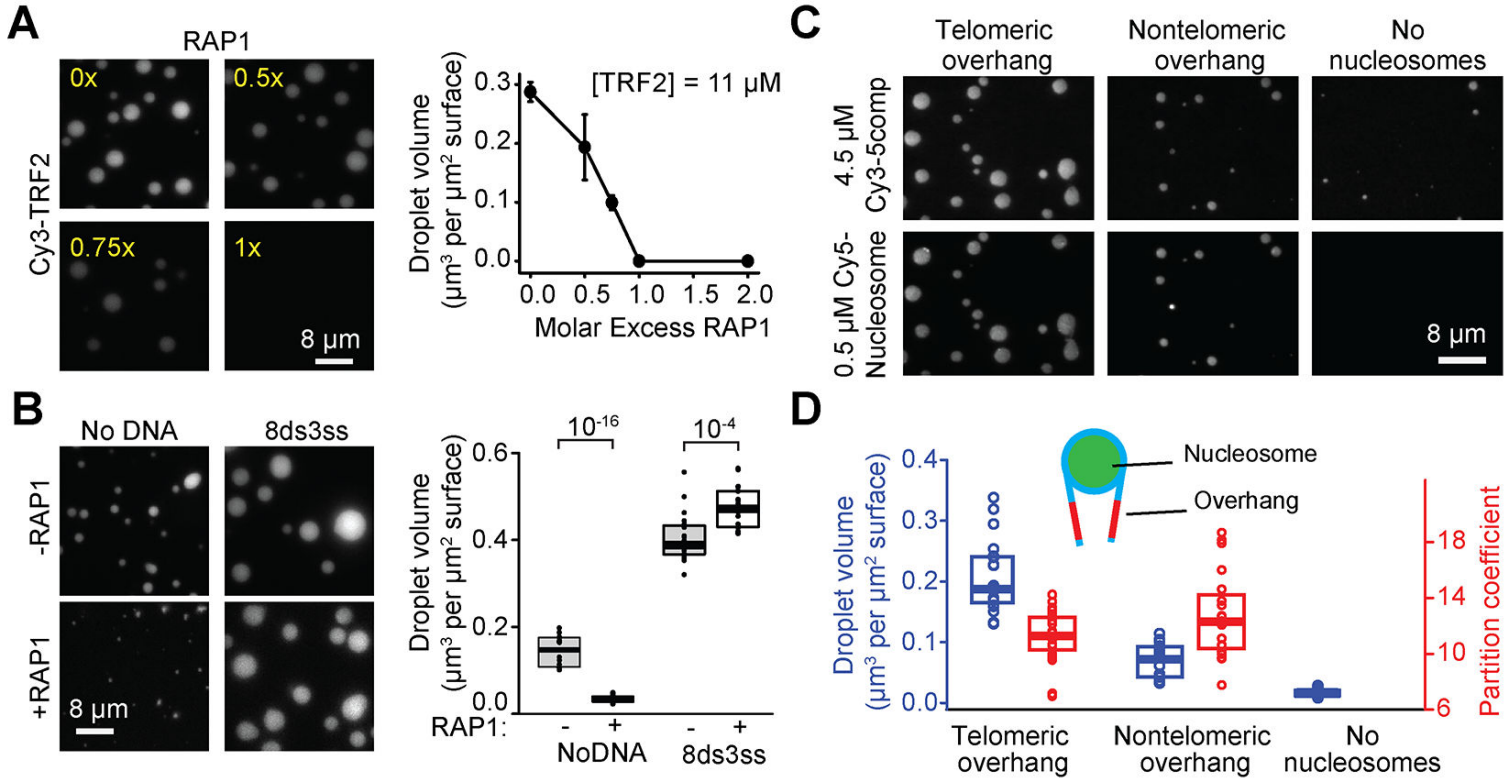
Telomere-associated proteins modulate phase separation of shelterin droplets. **A.** (Left) Increasing the molar ratio of RAP1 inhibits phase separation of TRF2 droplets. Droplets were formed in the presence of 2.5 μM 8ds3ss DNA. (Right) The total volume of TRF2 condensates settled per micron squared area on the coverslip as a function of RAP1 concentration (mean ± SD, n = 20 with two technical replicates). **B.** (Left) 5comp droplets formed with or without equimolar RAP1 and in the presence or absence of 2.5 μM 8ds3ss DNA. Complex concentration was set at 4.5 μM. (Right) The total volume of shelterin condensates settled per micron squared area on the coverslip in the absence or presence of RAP1. The center and edges of the box represent the median with the first and third quartile (n = 20 with two technical replicates). The p-values were calculated from a two-tailed t-test. **C.** Example images show phase separation of 4.5 μM 5comp in the presence and absence of nucleosomes wrapped with telomeric or nontelomeric DNA. **D.** Volume of droplets settled per micron squared area and partition coefficient of nucleosomes into 5comp droplets. The center and edges of the box represent the median with the first and third quartiles (n = 20 droplets with two technical replicates). See also Figure S6.

In mammalian cells, telomeres also associate with the sixth component of shelterin, RAP1, and nucleosomes, and we sought to examine their impact on telomeric phase separation *in vitro*. We found that RAP1 fully inhibits phase separation of TRF2 when mixed at an equimolar concentration (Figure 6A), presumably because RAP1 binding to the TRF2 hinge domain prevents this region to contribute to phase separation (Soranno et al., 2021). RAP1 also reduced the total volume of shelterin droplets without DNA when mixed at equal concentrations, and it moderately reduced the volume of 4comp2 droplets in the presence of DNA (Figures 6B and S6A-B). However, the addition of RAP1 only had a minor effect in the presence of telomeric DNA when all six shelterin subunits were present (Figure 6B), suggesting that the telomeric DNA scaffold and the interactions of the other shelterin subunits counteract RAP1’s inhibitory effect on phase separation.

We also purified mono-nucleosomes wrapped with Widom positioning DNA that contains either a telomeric or a nontelomeric overhang. We observed that mono-nucleosomes do not form liquid droplets on their own (Figure S6C) but are sequestered strongly into 5comp droplets in the absence of additional DNA (Figure 6C). The telomeric nucleosomes stimulated phase separation of 5comp, while less droplet formation occurred in the nontelomeric nucleosomes or buffer-only conditions (Figure 6D and S6D). These results indicate that heterotypic interactions between shelterin and telomeric DNA drive phase separation, even in the presence of other abundant factors like nucleosomes that localize to telomeres.

### Phase-separated shelterin selectively recruits associated factors.

**Figure 7.**
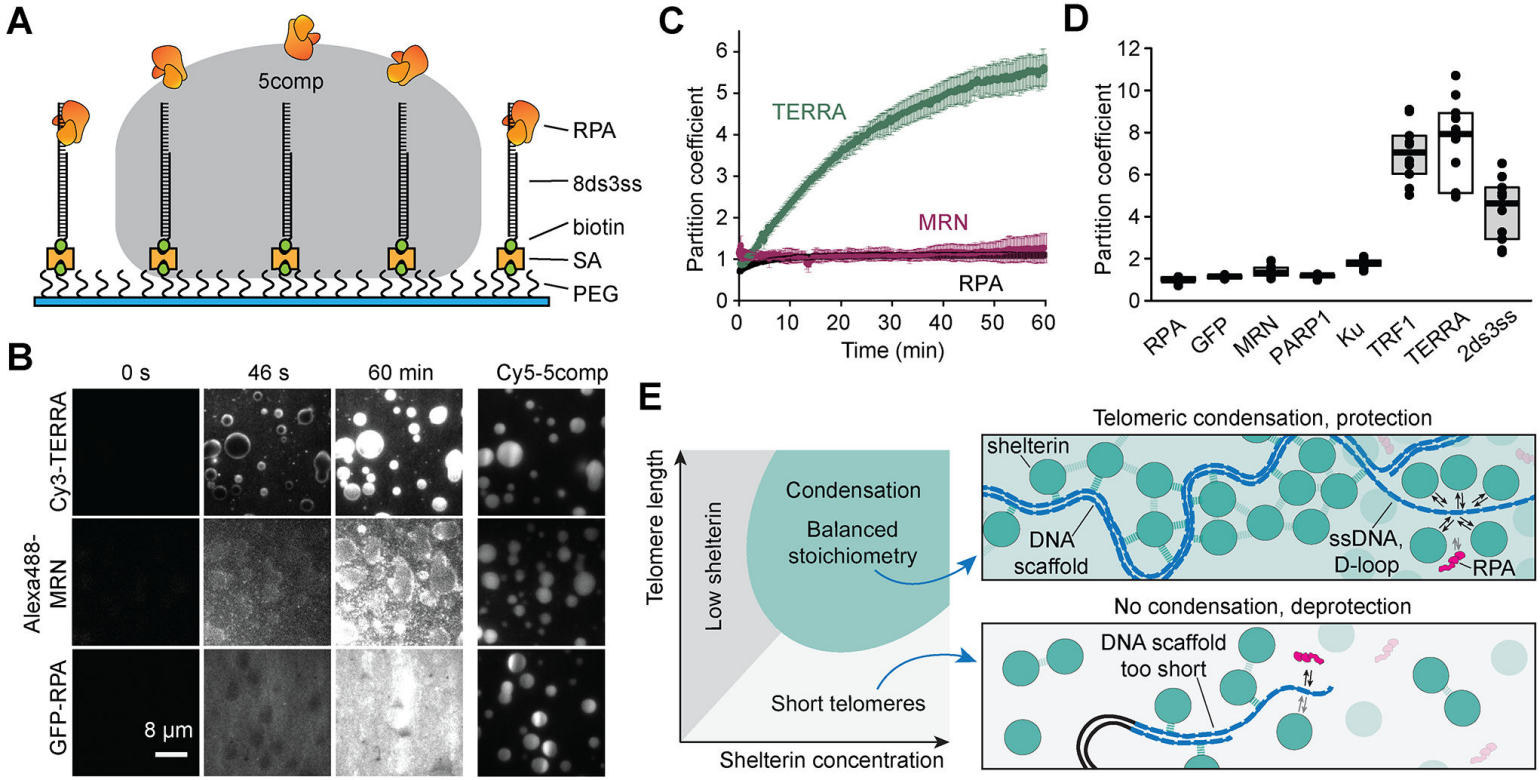
Shelterin droplets selectively recruit telomere-associated factors. **A.** 5comp droplets are settled onto PEG surfaces decorated with 8ds3ss. (PEG: polyethylene glycol, SA: streptavidin; not to scale). **B.** 100 nM Cy3-TERRA, 15 nM Alexa488-MRN complex, or 100 nM GFP-RPA are introduced to 7.6 μM Cy5-5comp droplets. TERRA partitions strongly into the droplets, while MRN and GFP-RPA are initially excluded from the droplets and uniformly distributed after 60 min incubation. **C.** Partitioning of 100 nM Cy3-TERRA, 15 nM Alexa488-MRN, or 100 nM GFP-RPA into 7.6 μM 5comp droplets over time (mean ± SD, n = 3 droplets per condition). **D.** Partition coefficients of DDR proteins and telomere-associated factors in 7.6 μM 5comp droplets after 60 min incubation. The center and edges of the box represent the median with the first and third quartile (n = 10 droplets per condition). **E.** (Left) Multicomponent phase diagram of telomere condensation with balanced stoichiometry. No condensation results at low shelterin concentrations and/or short telomeres. (Top) Telomere condensation, formed by both heterotypic (dark dashes) and homotypic (light dashes) interactions, selectively recruit telomere-associated factors while acting as a diffusion barrier against other components that target telomeric DNA, like RPA. The enrichment of shelterin, and thus POT1, outcompetes RPA binding to ssTEL. (Bottom) Shortened telomere scaffold cannot recruit enough shelterin to form a condensate, which could fail to protect the ssTEL overhang against RPA binding. See also Figure S7 and Movie S5.

To investigate selective permeability of shelterin droplets in an *in vitro* system that could mimic protection of the ssTEL overhang, we settled 5comp droplets onto surfaces coated with 8ds3ss (Figure 7A) and flowed fluorescently labeled queries into the chamber. When TRF1, telomeric noncoding RNA (TERRA) (Chu et al., 2017), or telomeric DNA were introduced into the chamber, they strongly partitioned into the droplets within a few minutes (Figures 7B-C and S7A), indicating that these settled droplets can accumulate favorable biomolecules. Consistently, TERRA partitioned less strongly into shelterin droplets containing TRF2^ΔB^ or TRF2^Acidic^ (Figure S7B), likely due to the loss of the interactions between TERRA and TRF2’s basic domain (Deng et al., 2009).

We then tested access of replication protein A (RPA), which activates the ATR pathway by binding to the ssTEL overhang (Denchi and de Lange, 2007; Gong and de Lange, 2010; Takai et al., 2011; Wold, 1997), and the Mre11-Rad50-Nbs1 (MRN) complex, which activates the ATM pathway at DNA double-strand breaks (DSBs) (Lamarche et al., 2010; Myler et al., 2017). Both GFP-RPA and Alexa488-MRN were distributed uniformly inside and outside the droplet after 10 min, rather than being enriched inside the droplet (Figure 7B-D, Movie S5). Downstream DDR factors PARP1, which is involved in the homologous recombination (HR) pathway (Rai et al., 2016), and the Ku70-Ku80 complex (Ku), which binds DSBs and is part of the non-homologous end joining (NHEJ) pathway (de Lange, 2018), also exhibited near-uniform partitioning inside and outside shelterin droplets (Figures 7D and S7C-E). Furthermore, GFP-RPA diffused into the droplets more slowly than telomere-associated factors and GFP (Figure S7A), suggesting that telomeric condensates could act as a diffusion barrier to biomolecules with a large Stokes radius (Frey and Gorlich, 2007; Wei et al., 2017). The addition of excess TRF1 or TRF2 did not speed up the diffusion of RPA (Figure S7F), suggesting that slow diffusion of RPA is not due to the high viscosity of 5comp droplets. However, RPA partitioned more strongly into 5comp shelterin containing TRF2^Acidic^ (Figure S7G), likely due to weakening of phase separation under these conditions. These results suggest that LLPS of shelterin selectively recruits and enriches telomere-associated factors independent of their size (Figure 7E).

## Discussion

In this study, we use *in vivo* and *in vitro* biophysical interrogation to demonstrate that telomeres represent a phase-separated liquid-like compartment. This compartment is formed through protein-protein and protein-DNA interactions, which give rise to the unique physicochemical properties of telomeres. We propose that the repetitive nature of telomeric DNA serves as a “super-scaffold” (Söding et al., 2020), effectively oligomerizing phase separation-prone proteins to drive the formation of a liquid compartment that protects the chromosome terminus (Fig 7E). In addition, our findings elucidate important organizing principles that likely underlie the formation of other genomic compartments.

Due to their constrained diffusion in mammalian cells, telomeres do not coalesce into a single condensate as would be expected in an equilibrium system. We speculate that random merger events would promote telomeric DNA end-to-end fusions and genome instability, and therefore the cell maintains telomeres as multiple distinct condensates. However, telomere clustering has been reported in TERT positive human cells (Adam et al., 2019) and ALT cells show fewer telomere puncta than the number of chromosome ends (Draskovic et al., 2009). It remains to be determined whether end-to-end fusion of telomeres is due to higher mobility or interactions with other phase separating condensates (such as APBs) in these cells. We repurposed the Corelet system to bring telomeres together on-demand, and showed that telomeres coalesce upon contact. This optogenetic method can be used to bring other chromatin loci together and could thus be a powerful approach to study the role of genomic compartmentalization in gene regulation and cellular function.

Previous examples of intracellular phase separation have primarily focused on the role of homotypic IDR-IDR interactions that behave as single component systems and exhibit a fixed *c*_sat_ (Nott et al., 2015; Wang et al., 2018). However, many phase separating systems utilize both homotypic and heterotypic interactions to form a complex network of multicomponent interactions (Alberti et al., 2019; Banani et al., 2016). Here, we show that the telomere is a multicomponent compartment whose formation relies on heterotypic interactions of the shelterin components and the scaffolding telomeric DNA (Riback et al., 2020). Consistently, synthetic oligomerization of shelterin components cannot form *de novo* condensates away from telomeres in living cells, except at exceedingly high concentration and valence.

Using an *in vitro* reconstitution approach, we found that the TRF1 and TRF2 subunits of human shelterin form liquid droplets, in agreement with an emerging study on *in vitro* phase separation of TRF2 (Soranno et al., 2021). Both IDRs and dimerization domains are required for TRF1 and TRF2 phase separation, and the differentially charged N-terminal domains are responsible for their distinct properties of condensation in the presence and absence of telomeric DNA. Consistent with our *in vivo* results, TRF1 and TRF2 together drive phase separation when in complex with other shelterin components and telomeric DNA.

Collectively, our results are consistent with a model of telomeres as condensed liquid compartments, in which shelterin components drive local condensation around the valence-amplifying super-scaffold of telomeric DNA. A balance between the length of telomeric DNA and the stoichiometry of the component factors affect the formation and composition of telomere condensation (Fig 7E). In accordance with this model, telomere function *in vivo* is controlled by protein levels of TRF1 and TRF2 (Celli and de Lange, 2005; Sfeir and de Lange, 2012; Ye et al., 2004) and the length of telomeric repeats (Galati et al., 2013; Shay and Wright, 2005). An imbalance of these factors may destabilize the structure and deprotect the telomere ends. Partial knockdown of TRF1 and TRF2 triggers several DDR pathways even though TRF1 and TRF2 are still abundant at these telomeres (Cesare et al., 2013; Orun et al., 2016). Additionally, as telomeres shorten in aging tissues, they fail to recruit sufficient shelterin to suppress DDR signals (Lackner et al., 2011). Consistent with these observations, we have shown that increasing the length of the telomeric DNA triggers phase separation at a lower concentration of TRF2 *in vitro.* Phase separation could also explain the mechanism of action of a dominant-negative allele of TRF2 within the context of the multicomponent network. This mutation is capable of dimerization with endogenous TRF2 but lacks DNA binding and the N-terminal domain, which may alter the interaction valence of the shelterin complex, leading to loss of compartmentalization and end-protection (van Steensel et al., 1998).

We found that *in vitro* shelterin droplets are more enriched with telomere-associated factors than the DDR proteins. These results suggest that through selective permeability the telomeric condensate could potentially recruit specific complexes like the DNA replication machinery and telomerase to the telomeric DNA, while limiting access of DDR factors during large-scale rearrangements of telomeric DNA. Selective partitioning of POT1, but not RPA, into shelterin droplets may also explain how POT1 can outcompete RPA binding to the displacement loop (D-loop) and the ssTEL overhang even though RPA is more abundant and has similar affinity for the ssTEL (Lazzerini-Denchi and Sfeir, 2016; Takai et al., 2011).

### Limitations of the Study

We present evidence that telomeres form liquid condensates using optogenetic manipulation in live cells and in vitro reconstitution of the shelterin complex. The phase separation model we propose is not mutually exclusive with the t-loop model, but provides an explanation for how shelterin compartmentalizes telomeric chromatin and regulates telomere function through selective recruitment of telomere-associated factors. The physiological relevance of LLPS and selective permeability for telomere end protection remain to be defined in vivo.

## STAR METHODS

### RESOURCE AVAILABILITY

#### Lead Contact

Further information and requests for resources and reagents should be directed to and will be fulfilled by the Lead Contact, Ahmet Yildiz (yildiz@berkeley.edu).

#### Materials Availability

All unique/stable reagents generated in this study are available from the Lead Contact upon request.

#### Data and Code Availability

This study did not generate any datasets/code amenable for depositing into public repositories. All data reported in this paper will be shared by the lead contact upon request. Any additional information required to reanalyze the data reported in this paper is available from the lead contact upon request.

### EXPERIMENTAL MODEL AND SUBJECT DETAILS

#### Cell Culture

Recombinant proteins were purified from Sf9-ESF *S. frugiperda* insect cells (RRID:CVCL_0549; female) grown at 27 °C in ESF 921 Insect Cell Culture Medium (Expression Systems, NC903611) supplemented with 1% fetal bovine serum (Corning, 35-010-CV) and 1% antibiotic-antimycotic (Thermo Fisher Scientific, 15240062). All human cell lines were incubated in and grown at 37°C with 5% CO_2_. U2OS cells were obtained from the ATCC and hTERT-RPE1 cells (p53 −/−, Rb −/−) were cultured in DMEM (GIBCO, 11995065) with 10% FBS (Atlanta Biological, S11150H) and 1% streptomycin and penicillin (GIBCO, 15140122), grown at 37°C with 5% CO_2_. The HeLa RMCE GFP-TRF1 cell line was cultured in DMEM with 10% FBS (Atlanta Biological, S11150H), 1% streptomycin and penicillin, and 1 μg/ml puromycin (Sigma, P7255). The U2OS cells were authenticated via ATCC's STR profiling. The other cell lines were not authenticated.

### METHOD DETAILS

#### Shelterin protein purification

Constructs for expressing individual components of the human shelterin complex were tagged with an N terminal ZZ affinity tag, TEV cleavage site, and YBBR labeling site and cloned into a Baculovirus vector. A construct expressing both TPP1 and TIN2 (TIN2 did not express on its own or without a solubility tag) and constructs expressing four- or five-component shelterin were cloned using a BigBac vector as described (Ferro et al., 2019). For the four-component shelterin BigBac construct, POT1 was given an N terminal YBBR tag, POT1 and TRF2 were each given an N terminal ZZ affinity tag and a TEV cleavage site, and TIN2 and TPP1 were each given an N-terminal His-MBP affinity tag and a TEV cleavage site. For a full list of constructed plasmids, see the Key Resources Table. Protein was purified from insect cells as previously described (Ferro et al., 2019). Briefly, plasmids containing genes of interest were transformed into DH10Bac competent cells (Berkeley MacroLab), and Bacmid DNA was purified using ZymoPURE miniprep buffers (Zymo Research, D4210) and ethanol precipitation.

Insect cells were transfected using Fugene HD transfection reagent (Promega, E2311). The virus was amplified in progressively larger cultures. 1 mL of the P1 virus was used to infect 50 mL of Sf9 cells at 1 million cells/mL for 72 h. 10 mL of the P2 virus was used to infect 1 L of Sf9 cells at 1 million cells/mL and expression proceeded for 72 h. Cells expressing the protein of interest were harvested at 4,000 *g* for 10 min and resuspended in 50 mL lysis buffer (50 mM HEPES pH 7.4, 1 M NaCl, 1 mM PMSF, 1 mM DTT, and 1 tablet of protease inhibitor (Sigma, 4693132001)). Lysis was performed using 15 loose and 15 tight plunges of a Wheaton glass dounce. The lysate was clarified using a 45 min, 360,000 *g* spin in a Ti70 rotor. The supernatant was incubated with 1 mL IgG beads (IgG Sepharose 6 Fast Flow, GE Healthcare, 17096902) for ZZ-tagged TRF1, TRF2, and POT1 constructs or 1 mL amylose beads (New England BioLabs, E8021S) for co-expressed TPP1 and TIN2 and shelterin constructs for 1 h. Beads were washed with 40 mL of labelling buffer (50 mM HEPES pH 7.4, 300 mM NaCl, 10 mM MgCl_2_, 1 mM EGTA, 10% glycerol, 1 mM DTT). Beads were then collected and incubated with purified SFP protein (Addgene #75015)and a fluorescent dye functionalized with CoA (Lumidyne, custom synthesis) at room temperature for 30 min. Beads were washed in 40 mL labeling buffer, collected, and incubated with TEV protease (Berkeley Macrolab, Addgene #8827) for 1 h at room temperature to elute the protein. For shelterin protein preps, the protein was additionally incubated with 0.3 mL Ni-NTA beads (HisPur, Thermo Fisher Scientific, 88221) in 20 mM imidazole to remove the His-MBP and TEV in solution. After 30 min of incubation at °C, the beads were pelleted and the unbound protein was collected from the supernatant.

For the TRF1 and TRF2 mutant proteins, all purification steps were carried out in 1M NaCl to prevent aggregation. After TEV cleavage, the mutant proteins were concentrated and resuspended to reduce NaCl concentration to 300 mM. Finally, the protein was concentrated using Amicon Ultra 30K concentrators, concentration was measured using Bradford reagent (Bio-Rad, 500-0006), and aliquots were snap-frozen in liquid nitrogen. Isoelectric points were calculated with ExPASy ProtParam.

4comp2 was purified using the BigBac system, where subunits were coexpressed from the same vector. 4comp1 was created by mixing known concentrations of purified TRF1, coexpressed TPP1 and TIN2, and POT1 on ice. 5comp consisted of purified 4comp2 mixed with purified TRF1. 4comp1 and 4comp2 were run through a Superdex 200 Increase 10/300 GL size exclusion column (Cytiva, 28-9909-44) to separate assembled complexes from individual proteins and subcomplexes. Mixing purified proteins on ice produced results comparable to coexpressing the components (Figure S4G-H).

#### DDR protein purification

GFP-RPA was purified as previously described (Schaub et al., 2018). GFP was expressed in Rosetta cells (Berkeley MacroLab) using the GFP plasmid (Addgene 54762). This culture was added to 1 L of LB media and grown for 3 h until OD_600_ reaches 0.7. Cells were induced with 0.2% L-arabinose and incubated for 4.5 h at 37 °C in a shaker. After harvesting cells at 4,785 *g* for 15 min in a JLA 8.1 rotor, 500 mL cell pellets were incubated with 40 mL lysis buffer (50 mM HEPES pH 7.4, 300 mM NaCl, 20 mM imidazole, 1 mM PMSF, 1 mM DTT, and 1 tablet of protease inhibitor (Sigma, 11836170001)). Cells were lysed with a sonicator and spun in a Ti70 rotor at 117,734 *g* for 30 min. The supernatant was incubated with 2 mL of washed Ni-NTA beads (HisPur, Thermo Scientific, 88221) for 1 h at 4 °C. Beads were collected in a Bio-Rad column and washed in lysis buffer. Protein was cleaved off the beads with TEV protease at room temperature for 1 h and concentrated in an Amicon Ultra 10K concentrator. Protein concentration was measured using Bradford reagent (Bio-Rad, 500-0006). Protein was aliquoted and snap-frozen in 10% glycerol.

Mre11-Rad50-Nbs1 (MRN) complex and Ku were purified from Sf21 insect cells as previously described (Myler et al., 2017), with the addition of a 3x FLAG tag on the C-terminus of Mre11 and a 3x HA tag on the C-terminus of Ku80. PARP-1 was purified from Rosetta *Escherichia coli* cells as previously described (Caron et al., 2019; Langelier et al., 2011), with the addition of an N-terminal His-SUMO-HA tag. To label MRN, anti-FLAG (Sigma-Aldrich, F1804) was labeled using an Alexa Fluor 488 antibody labeling kit (Thermo Fisher Scientific, A20181). Alexa488 anti-FLAG at 3x molar excess was then incubated with the MRN complex on ice for 10 minutes. Ku and PARP-1 were labeled by incubating 2 hr at room temperature with 5-fold molar excess maleimide-coupled Atto488 dye (Sigma-Aldrich, 28562). Free dye was removed using a 40kDa Zeba spin desalting column (Thermo Fisher Scientific, 87766).

#### Nucleosome preparation

Histones were expressed from pET-H2A, pET-H2B, pET-H3, and pET-H4 constructs (Luger et al., 1999) in BL21(DE3)pLysS cells (Sigma-Aldrich, 69451) and purified according to previously described procedures (Dyer et al., 2004). Inclusion bodies were solubilized in DMSO and unfolding buffer (7M Guanidine HCl, 20 mM Tris-HCl, pH 7.5, 10 mM DTT) and purified using anion exchange chromatography (HiTrap SP HP, GE Life Sciences, 95056-076) in SAU buffer (7M deionized urea, 20 mM sodium acetate pH 5.2, 5 mM beta-mercaptoethanol (BME), 1 mM EDTA), eluting with a salt gradient from 0.2 to 0.6 M NaCl. Following dialysis of the peak fractions overnight in water + 2 mM BME, histones were buffer exchanged into unfolding buffer and concentrated. Octamers were assembled by mixing equimolar amounts of each histone and dialyzing overnight in refolding buffer (2M NaCl, 10 mM Tris pH 7.5, 1 mM EDTA, 5 mM BME). Next, octamers were purified in refolding buffer with a Superdex 10/300 GL column (GE Life Sciences, 17517501) using an Akta Pure chromatography system. Octamer formation in peak fractions was verified by Coomassie staining of SDS-PAGE gels. Nucleosome DNA sequences were amplified with Phusion polymerase (New England BioLabs, M0530L) from pGEM-3z/601 (Addgene plasmid #26656) (Lowary and Widom, 1998) using the following primer pairs for the standard nucleosome: 5’-Cy5-CTGGAGAATCCCGGTGCCG-3’ and 5’-ACAGGATGTATATATCTGACACG-3’, and for the telomere-tagged nucleosome the primer pair 5’-Cy5-TCGAATTCTTAGGGTTAGGGTTACCCTGGAGAATCCCGGT-3’ and 5’- CTGGATCCTAACCCTAACCCTAAGCACAGGATGTATATATCTGA-3’ were used. Oligonucleotides were synthesized by IDT. PCR products were verified on SYBR-Safe (Thermo Fisher Scientific, S33102) stained gels and purified using the Genejet PCR purification kit (Thermo Scientific, K0701). Nucleosomal core particles (NCPs) were then reconstituted by mixing the DNA and octamer at a molar ratio of 1.1:1 and slowly dialyzing into low salt, according to the procedure of Chua et al. (Chua et al., 2016) except that the dialysis was stopped at 0.1 M KCl. NCPs were purified on a HiTrap DEAE-FF (Cytiva, 17515401) column, first binding to the column in TCS buffer (20 mM Tris, pH 7.5, 1 mM EDTA, 1 mM DTT) and then eluting with an increasing gradient of KCl in TES buffer (10 mM Tris, pH 7.5, 0.5 mM EDTA). Peak fractions were examined on 0.5% agarose gels for Cy5 fluorescence (Amersham Typhoon; GE Life Sciences, 29238583). Fractions containing NCPs were pooled and dialyzed overnight in TCS buffer, then concentrated using Millipore centrifugal filters.

#### Formation and labeling of DNA substrates

For all DNA substrates except 39ds0ss, ssDNA sequences were ordered from IDT. Solutions of equimolar complementary sequences suspended in annealing buffer (10 mM Tris pH 7.5, 50 mM LiCl) were mixed and incubated in a hot plate at 95 °C for 5 min. The sample was then removed from the hot plate and allowed to cool to room temperature over 2 h. Comparing the molecular weights of the ssDNA oligos with that of the annealed dsDNA on an agarose gel confirms that annealing efficiency is high (Figure S2C). The 39ds0ss substrate was created by PCR amplifying a region of telomeric repeats from a plasmid, and the purity and length of the resulting DNA was verified on a 0.8% agarose gel. DNA was Cy3 labeled using a Label IT kit (Mirus Bio, MIR 3600).

#### Imaging of condensates

Slides were incubated with wash buffer (50 mM HEPES pH 7.4, 150 mM NaCl, 10 mM MgCl_2_, 1 mM EGTA, 1 mM DTT, 1% pluronic) for 5 min. Samples were settled onto the coverslip for 25 min before imaging. Imaging was performed using a Nikon Ti-E Eclipse microscope equipped with a 100X 1.49 N.A. Plan Apo oil immersion objective (Nikon). The samples were excited in near-TIRF using 488, 561, and 633 nm laser beams (Coherent). The emission signal was passed through a filter wheel and detected by Andor Ixon EMCCD Camera (512x512 pixels). The effective pixel size was 106 nm after 1X magnification and 160 nm after 1.5X magnification. Image processing is described in the “Quantification and Statistical Analysis” section.

For experiments involving DNA bound to the surface of the slide, chambers were incubated with 1 mg/mL Biotin-BSA (Sigma-Aldrich, 9048-46-8) for 2 min, incubated with 1 mg/mL streptavidin (Thermo Fisher Scientific, 434301) for 2 min, washed twice with 20 μL wash buffer, incubated with 1 μM biotinylated 8ds3ss DNA (IDT) for 2 min and washed twice with 20 μL wash buffer. Shelterin droplets were formed in a test tube, flowed into the chamber, and settled on the coverslip for 10 min. 5 μL solution containing the protein or DNA being tested was introduced to the chamber, and the sample was imaged using time-lapsing for 1 s every 10 s for 1 hr.

#### Cell culture

All DNA fragments of interest were PCR-amplified using Phusion High-Fidelity DNA Polymerase (New England BioLabs, M0530L). The hTRF1 and hTRF2 gene fragments were synthesized by IDT as gBlocks, with synonymous codon optimization to reduce repetitive DNA tracts. These fragments and point mutants were cloned into a linearized FM5 lentiviral vector. FM5 lentiviral vectors carried standardized linkers to insert the PCR fragments using the In-Fusion HD cloning kit (Takara Bio, 638910) (Sanders et al., 2020). Corelet constructs, unless otherwise noted, were cloned into the pHR lentiviral vector and confirmed by GENEWIZ Sanger sequencing.

Lentiviruses were generated by plating Lenti-X 293T cells (Takara Bio, 632180) into 6-well plates to reach ~70% confluence at the time of transfection. 24-36 hours after plating the Lenti-X cells, the transfer plasmid (1.50 μg), pCMVdR8.91 (1.33 μg), pMD2.G (0.17 μg) were transfected into the cells using FuGENE HD incubated in OptiMEM (modified from (Shin et al., 2018)). Transfer plasmids for the 53BP1 counting assay were transfected into Lenti-X cells with the helper plasmids VSVG and PSP with the Transit293 transfection reagent (Mirus, MIR 2700), following the protocol listed in Sanders et al. 2014 (Sanders et al., 2020). The supernatant-containing viruses were harvested 48 hours after transfection and filtered with a 0.45 μm filter (Pall Life Sciences), then used immediately or stored at −80°C. U2OS and hTERT-RPE1 cells plated at low (10-20%) confluency in 96-well glass-bottom plates (Cellvis) were transduced for 2-3 days before the washout of the virus, replacement with fresh media, and subsequent live-cell imaging experiments. Virus used for the formation of TRF1-mCh-sspB droplets away from telomeres at exceedingly high TRF1 concentrations was concentrated 10x using the Lenti-X Concentrator (Takara Bio, 631231), following the manufacturer’s protocol.

#### Live cell imaging

Cells plated on 96-well glass-bottom plates were incubated at 37°C and 5% CO_2_ by an Okolab microscope stage incubator with 96-well insert during all imaging experiments. Confocal microscopy was performed on a spinning disk (Yokogawa CSU-X1) confocal microscope with an Andor DU-897 EMCCD camera on a Nikon Eclipse Ti body using a 100x oil immersion Apo TIRF objective (NA 1.49). The following wavelength lasers were used to image the respective constructs: constructs with mGFP (488 nm), mCherry (561 nm), miRFP (640 nm). Fixed samples in the 53BP1 counting assay also used the 405 nm laser to detect nuclei stained with Hoechst (Thermo Fisher Scientific, H3570) or DAPI (Vectashield, H-2000-10).

#### Estimation of telomere component concentration *in vivo*

Both estimate that there are on the order of thousands of TRF2 dimers in a cell. Telomeric puncta are estimated to be 60-300 nm in diameter, and because virtually all TRF2 localize at telomeres (Palm and de Lange, 2008), the local concentration of dimers within TRF2 puncta would be hundred-micromolar.

#### FRAP assays

FRAP experiments were performed on a Nikon A1 laser scanning confocal microscope using a 60x oil immersion objective. A single telomere marked by shelterin proteins of interest was photobleached with the 488 nm and 640 nm laser each at bleaching power of ~ 400 kW cm^-2^. The cell was imaged every 2 s for 10 s of pre-bleach, and every 2 s post-bleach for 200 s. FRAP data were normalized by using the normalization method (Day et al., 2012). The first post-bleach point was set to zero.

#### Mean Squared Displacement measurements

Time-lapse movies were taken of U2OS cells with GFP-TRF1 or FUS_N_-miRFP-TRF1 overexpression and HeLa RMCE GFP-TRF1. Cells were plated 24 h before imaging on 96-well glass-bottom plates coated with fibronectin to reach high confluency. Each movie was 1 h long, imaging 1 s per frame.

#### Optogenetic telomere coalescence

Local activation was performed by using a Mightex Polygon digital micromirror device (DMD) to pattern blue light (488nm) stimulation from a Lumencor SpectraX light engine using Nikon Elements software. U2OS cells expressing the optogenetic telomere coalescence constructs FUS_N_-miRFP-TRF1, NLS-GFP-iLId-Fe and FUS_N_-mCherry-sspB were imaged using a specific local activation protocol, as follows. Pre-activation, imaging the mCherry (541 nm beam) and miRFP (640 nm beam) channels every 5 s for 15 s. Activation, wherein an elliptical region of interest (ROI) was used to locally activate two telomere foci to nucleate and grow FUS_N_ Corelet droplets using the 485 nm DMD laser every 5 s for 6 min. A second activation sequence used a smaller, circular ROI aimed at the junction between two FUS_N_ Corelet droplets every 5 s for 4 min to encourage them to fuse. Finally, the FUS_N_ droplet was deactivated for 10 min by only imaging the mCherry and miRFP channels every 5 s, which allows the droplets to dissolve and pull together any attached telomeres. The second set of telomere coalescence constructs (iLId-miRFP-TRF1) uses a similar local activation protocol but only a single circular activation ROI for 3 min and a longer deactivation sequence (15-30 min).

#### Corelet experiments

TRF1^WT^, TRF2^WT^, and TRF1 mutant Corelet experiments were imaged every 5 s and followed this protocol: 15 s pre-activation (561 and 640 nm lasers) and 10 min of activation for local activation (488, 561, and 640 nm lasers). Each locally activated telomere and region away from telomere was normalized by subtracting the background from the ROI and divided by the average intensity of all other telomeres in the same cell minus the background. The first and last frames of activation were quantified.

#### siRNA TRF2 knockdown

Endogenous TRF2 levels were knocked down using siRNAs made by IDT with sequences from Takai et al. 2003 (Takai et al., 2003) and Yang et al. 2015 (Yang et al., 2015). siRNA transfection efficiency was estimated by transfecting a Cy3 labeled control siRNA (ThermoFisher Scientific, AM4621). To quantify transfection efficiency, three biological trials were transfected, fixed, stained with Hoechst or DAPI, and imaged. Nuclei were segmented with DAPI/Hoechst channel in FIJI and the intensity of Cy3 within each nucleus was recorded. Background intensity was subtracted from each, and the percent of cells with Cy3 signal at least 200 A.U. above background was plotted. The 1x condition was used for the 53BP1 counting assay. TRF2 knockdown efficiency was then validated by western blots (Figure S5B).

#### Western blot analysis

hTERT-RPE1 cells were plated on 6-well plates 24 hrs before siRNA treatment, and cells with or without siRNA treatment (siRNA #2 from Takai et al., 2003 (Takai et al., 2003) and a control scramble siRNA from Yang et al., 2015 (Yang et al., 2015) were grown for 48 hr before harvesting. Cell pellets were resuspended in 300ul RIPA buffer (BCA, 89901) with protease inhibitor (Sigma Aldrich, 4693132001). 1ul of benzonase (Sigma Aldrich, E1014-25KU) was added to each sample and left on ice for 30 min, each sample was spun down for an additional 30 min at 4°C, 30ul of lysate was resuspended in sample buffer (Thermo Fisher, NP0007), boiled at 100°C for 5 min with 15ul of the mix loaded for SDS-PAGE. Samples were run on a NuPAGE 4-12% Bis-Tris protein gel (Thermo Fisher, NP0322BOX) and transferred onto Trans-Blot Turbo Mini 0.2 um PVDF transfer pack (Bio-Rad, 1704156) for 30 min. Membranes were blocked for 2 hr with 5% NFDM in 1X TBST (Fisher Scientific, AAJ62938K2), and incubated in block with the anti-TRF2 antibody 1:2000 (Novus Biologicals, NB110-57130) and anti-Histone H3 antibody 1:2000 (Abcam, ab10799) for the loading control overnight at 4°C. Membranes were washed three times 5 min each with 1X TBST, incubated with either the Peroxidase AffiniPure Goat anti-mouse IgG 1:10,000 (Jackson ImmunoResearch, 115-035-062) or the Peroxidase AffiniPure Goat anti-rabbit IgG secondary antibodies 1:10,000 (JacksonImmunoResearch, 111-035-144) for 30 min at room temperature. Membranes were washed three times 5 min each with 1X TBST and developed using the SuperSignal West Pico PLUS Chemiluminescent Substrate (Thermo Fisher, 34577), following the manufacturer’s protocol. To determine the knockdown efficiency, the background intensity was subtracted from each band intensity, then normalized relative to loading control and plotted as a ratio relative to scrambled RNAi (set at 100% for each trial).

#### 53BP1 foci counting assay

The siRNA #2 from Takai et al., 2003 (Takai et al., 2003) and a control scramble siRNA from Yang et al., 2015 (Yang et al., 2015) were used for the 53BP1 counting assay that used an IF-FISH protocol adapted from both the de Lange lab’s IF-FISH protocol and PNA bio’s FISH protocol. hTERT-RPE1 cells plated on glass-bottom 96 well plate 24 hr before transfection and were transfected twice with Oligofectamine (Thermo Fisher, 12252011) according to the manufacturer’s protocol (the second transfection was 24 hr after the first). Cells were transduced with 50-70ul of the rescue construct lentiviruses simultaneously with the siRNA treatment. Cells were then fixed with 4% paraformaldehyde for 5 min 48 hours after the first siRNA transfection, washed three times 5 min each with 1X TBST, and permeabilized for 15 min in 0.5% Triton X-100 buffer, incubated with block (10% goat serum and 0.1% Triton X-100 in 1X TBST) at room temperature for 1 hr, and incubated in block with anti-53BP1 antibody 1:50 (Novus Biologicals, NB100-305) overnight at 4°C. Cells were then washed four times 5 min each with 1X TBST, incubated with Goat anti-Rabbit IgG secondary antibody conjugated to Alexa fluor 647 (Thermo Fisher, A-21245) for 2 hours, washed four times, and fixed again with 4% paraformaldehyde. After three washes of 5 min each with 1X TBST, cells were dehydrated in 70%, 85%, 100% cold ethanol for 5 min each, air-dried for 15 min, denatured for 10 min with the hybridization buffer at 80°C. The hybridization buffer contained 70% formamide, 0.5% blocking reagent (Millipore Sigma, 11096176001), 20mM Tris-HCl pH 7.5, and a FITC labeled C-rich telomere probe (PNA bio, F1009). The samples were then incubated in the dark at room temperature for 2 hr, washed twice with 70% formamide, 10mM Tris-HCl pH 7.5 for 15 min each, washed three times for 5 min each with 1X TBST, left to air dry before mounting in Vectashield Plus Antifade Mounting Medium with DAPI (H-2000-10). 2x2 tiled images were taken from 31 z-stacks of 0.2um spacing on a spinning disk (Yokogawa CSU-X1) confocal microscope. 3D Objects Counter (Bolte and Cordelières, 2006) and 3D Multicoloc included in the 3D ImageJ suite (Ollion et al., 2013) were used to detect the nuclei, 53BP1, telomeres, and the number of colocalizations per stack. 3D segmented data was parsed in Python 3.7.10 with a custom Python script that counts the number of 53BP1 foci per nucleus.

#### miRFP-TRF2 dilute phase vs. total concentration

U2OS cells were transduced with 50 μl of miRFP-TRF2 lentivirus to result in differential overexpression levels. Images were taken from 11 z-planes of 0.5 μm spacing. Total concentration was calculated by taking the average intensity of the miRFP-TRF2 signal in an entire segmented nucleus, and dilute phase calculated by taking the average intensity of miRFP-TRF2 in the nucleoplasm, with bright telomeres masked out. *c*_dil_ is measured as the ‘background’ concentration when a condensed phase is present. In a single-component phase separating system, *c*_dil_ will saturate at a single ‘saturation concentration’ (*c*_sat_), while in a multicomponent phase separating system, *c*_dil_ may vary as a function of total system concentration.

### QUANTIFICATION AND STATISTICAL ANALYSIS

#### In vitro droplet image processing

To calculate the saturation concentration (*c*_sat_) of the proteins, droplets were identified using the Phansalkar function in Fiji (ImageJ 1.52p) with a 30-pixel radius and a minimum condensate size of 10 pixels. The volume of the condensates was estimated from 2D projections by taking the semi-principal axis in the z-plane as the geometric average of semi-principal axes in the XY plane. The total volume of the condensates settled per micron squared on the coverslip was quantified. Conditions that resulted in measurable condensate volumes were fit to linear regression in Origin. The x-intercept of the linear regression represents *c*_sat_, the minimum protein concentration that results in condensate formation. *c*_sat_ for TRF2 in the presence of different DNA constructs (Figure 3D) was determined as the lowest protein concentration for which condensates are visible (Figure S2D). The aspect ratio was calculated using the Phansalkar function with a 30-pixel radius to detect particles and the Fit Ellipse function to determine the major axis length by the minor axis length in Fiji. Fusion times were calculated as the time between the last frame where two droplets appear separated (i.e. no overlap) and the first frame where the fused droplet appears spherical (aspect ratio ~ 1).

#### Image segmentation for time-lapse imaging of telomeres

All images were analyzed in Fiji (ImageJ 1.52p) and MATLAB 2019b (Mathworks). The first frame of each movie was used to calculate inter-telomere spacing. Briefly, nuclei were segmented using Otsu’s method; telomeres were then segmented by filtering using an LoG (Laplacian of Gaussians) kernel and applying a two standard deviation threshold. Average pairwise distance and nearest neighbors were then calculated (*pdist2*) based on the weighted centroids of all telomeres within each nucleus (extracted from the punctate mask *regionprops*). The local concentration of TRF2 at telomeres was estimated to be 400 μM from the measured radius of telomeres (~100 nm, (Bandaria et al., 2016; Jeynes et al., 2017)) and estimated number of TRF2 at each telomere in cells (~1,000 on average) from immunoblotting (Takai et al., 2010) and superresolution imaging assays (Bandaria et al., 2016).

#### Mean Squared Displacement analysis

To analyze telomere movement, images were registered to correct for whole-cell movement using StackReg plugin in Fiji. Then, Trackmate was used to track telomere movement with subpixel resolution using a Laplacian of Gaussian detector and object diameter of 500 nm. Trajectories of telomeres were then created using LAP tracking with maximum linking and gap-closing distances of 500 nm and zero-gap frames. Trajectories were only used if they spanned at least half the number of frames of the movie, then coordinates exported to MATLAB to calculate mean squared displacement.

#### Integrated intensity predictions and measurements

Telomeres were segmented using an LoG filter threshold method. Their respective total integrated intensity was calculated by summing over the intensity per pixel in the identified region. Since the integrated intensity should be directly proportional to the volume (Berry et al., 2015), the average integrated intensity of each telomere was calculated pre-coalescence (defined as all frames wherein two puncta were identified) and summed; the error was estimated and propagated by taking the standard error of the mean over pre-coalescence frames. The resulting summed integrated intensity and error bar were used as the independent prediction of the integrated intensity post-coalescence (defined as all frames wherein only one object was detected).

#### Statistical analysis

Statistics for the Corelet experiments and 53BP1 counting assay were performed using GraphPad PRISM version 9.1.0 software (GraphPad). Statistical significance (when reported) was calculated by one-way ANOVA with multiple comparisons or two-tailed t-test as noted in the figure legends. Number of replicates, size of n and precision measures (mean, median, ± SE and ± SD) are noted in the figure legends and captions.

## Supplementary Material

Supplement

Movie S1 **Movie S1. Telomeres rarely coalesce due to their suppressed diffusivity in living cells; related to** Figure 1. Time-lapse movie showing that telomeres marked by GFP-TRF1 in HeLa cells are mostly stationary due to their subdiffusive motion.

Movie S2 **Movie S2. Specific local activation protocol using the Corelet system allows observation of liquid-like telomere coalescence in living cells; related to** Figure 2. (Left) The shrinking of FUS_N_ Corelet droplets pull in FUS_N_-miRFP-TRF1 marked telomeres to coalesce into a single telomere condensate. (Right) Telomeres relax back to more distal positions after detaching from FUS_N_ droplets.

Movie S3 **Movie S3. Telomere coalescence is not due to FUS_N_-FUS_N_ interactions as iLID fused to TRF1 demonstrates liquid-like telomere merging events; related to** Figure 2. iLID-miRFP-TRF1 acts as a seed only when it is bound to FUS_N_-mCherry-sspB upon light activation. The FUS_N_ droplet shrinks and pulls in the telomeres to merge upon light deactivation once FUS_N_-mCherry-sspB is completely dissolved.

Movie S4 **Movie S4. The fusion of TRF2, TRF1, and 5comp droplets in the presence of telomeric DNA; related to** Figures 3 and 5. TRF2 and TRF1 droplets containing DNA fuse within 30 seconds, on average, while 5-component shelterin droplets containing DNA adhere and relax but do not become spherical over several hundred seconds. Droplets were imaged with a TIRF microscope at 4.3 frames/s. Droplets were formed with 2.5 μM 8ds3ss telomeric DNA using either 22 μM TRF2, 44 μM TRF1, or 4.5 μM 5comp.

Movie S5 **Movie S5. RPA and TERRA diffuse into 5-comp droplets settled onto a DNA-coated slide; related to** Figure 7. Droplets formed from 5.3 μM 5comp in physiological salt were settled onto a slide coated with 8ds3ss telomeric DNA for 10 min before 100 μM GFP-RPA and 100 μM 60nt-TERRA were flowed onto the slide. RPA is distributed uniformly across the surface over several minutes, while TERRA partitions strongly into the shelterin droplets. TIRF imaging began 55 s after the introduction of RPA and TERRA to the sample. Time-lapse images were acquired for 200 ms every 10 s.

## Figures and Tables

**Table:** KEY RESOURCES TABLE

REAGENT or RESOURCE	SOURCE	IDENTIFIER
**Antibodies**		
Rabbit anti-TRF2	Novus Biologicals	Cat#NB110-57130
Mouse anti-Histone H3	Abcam	Cat#Ab10799
Anti-Mouse IgG, Peroxidase Conjugated, Goat	Jackson ImmunoResearch	Cat#115-035-062
Anti-Rabbit IgG, Peroxidase Conjugated, Goat	Jackson ImmunoResearch	Cat#111-035-144
Rabbit anti-53BP1	Novus Biologicals	Cat#NB100-305
Goat anti-Rabbit IgG, AlexaFluor 647 conjugated	Thermo Fisher Scientific	Cat#A-21245
Anti-FLAG M2, mouse	Sigma-Aldrich	Cat#F1804
		
		
		
**Bacterial and Virus Strains**		
XL1Blue	MacroLab, University of California Berkeley	N/A
Rosetta	MacroLab, University of California Berkeley	N/A
DH10Bac	MacroLab, University of California Berkeley	N/A
BL21(DE3)pLysS	Sigma-Aldrich	Cat#69451
		
**Biological Samples**		
		
		
		
		
		
		
**Chemicals, Peptides, and Recombinant Proteins**		
Fugene HD Transfection Reagent	Promega	Cat#E2311
TEV protease	MacroLab, University of California Berkeley	Addgene Cat#8827
Streptavidin	ThermoFisher Scientific	Cat#434301
LD655-CoA	Lumidyne Technologies	Custom synthesis
LD555-CoA	Lumidyne Technologies	Custom synthesis
Bradford Reagent	Bio-Rad	Cat#500-0006
Benzonase nuclease	Sigma Aldrich	Cat#E1014-25KU
Blocking reagent	Millipore Sigma	Cat#11096176001
Fetal Bovine Serum, Premium, Heat-Inactivated	Atlanta Biologicals	Cat#S11150H
Fibronectin Bovine Plasma	Millipore Sigma	Cat#F1141
FITC-TelC, C-rich telomere probe, FITC labeled	PNA bio	Cat#F1009
Formamide (Deionized)	Thermo Fisher	Cat#AM9342
GIBCO DMEM, High Glucose, Pyruvate	Thermo Fisher Scientific	Cat#11995065
GIBCO DPBS, no calcium, no magnesium	Thermo Fisher Scientific	Cat#14190144
GIBCO Opti-MEM Reduced Serum Medium	Thermo Fisher Scientific	Cat#31985062
GIBCO Penicillin-Streptomycin (10,000 U/mL)	Thermo Fisher Scientific	Cat#15140122
Hoechst 33342, 10mg/mL	Thermo Fisher Scientific	Cat#H3570
In-Fusion HD Cloning Plus	Takara Bio	Cat#638910
Lenti-X Concentrator	Takara Bio	Cat#631231
Normal Goat Serum Blocking Solution	Vector Laboratories	Cat#S-1000-20
NuPAGE™ LDS Sample Buffer (4X)	Thermo Fisher	Cat#NP0007
Oligofectamine	Thermo Fisher	Cat#12252011
Paraformaldehyde (16%)	Electron Microscopy Science	Cat#15710
Phusion® High-Fidelity DNA Polymerase	New England Biolabs	Cat#M0530L
Pierce RIPA buffer	BCA	Cat#89901
Protease Inhibitor tablets (EDTA-free)	Sigma	Cat#4693132001
Puromycin dihydrochloride from Streptomyces alboniger	Sigma	Cat#P7255
SuperSignal West Pico PLUS Chemiluminescent Substrate	Thermo Fisher	Cat#34577
Transit293 Transfection Reagent	Mirus	Cat#MIR 2700
TRIS-buffered saline (TBS, 10X) pH 7.4	Fisher Scientific	Cat#AAJ62938K2
Triton X-100	Promega	Cat#H5142
Tween-20	Thermo Fisher	Cat#BP337-100
Vectashield Plus Antifade Mounting Medium with DAPI	Vectashield	Cat#H-2000-10
ESF 921 Insect Cell Culture Medium	Expression Systems	Cat#NC903611
Antibiotic-antimycotic	Thermo Fisher Scientific	Cat#15240062
Fetal Bovine Serum	Corning	Cat#35-010-CV
ZymoPURE miniprep kit	Zymo Research	Cat#D4210
Alexa Fluor 488 antibody labeling kit	Thermo Fisher Scientific	Cat#A20181
Atto488 maleimide dye	Sigma-Aldrich	Cat#28562
SYBR-Safe	Thermo Fisher Scientific	Cat#S33102
Genejet PCR purification kit	Thermo Fisher Scientific	Cat#K0701
Cy3 Label IT kit	Mirus Bio	Cat#MIR 3600
Biotin-BSA	Sigma-Aldrich	Cat#9048-46-8
Streptavidin	Thermo Fisher Scientific	Cat#434301
		
		
		
		
		
**Deposited Data**		
		
		
		
		
**Experimental Models: Cell Lines**		
SF9-ESF S Frugiperda	Berkeley Cell Culture Facility	RRID:CVCL_0549
Human: U-2 OS	ATCC	ATCC® HTB-96™
Human: Lenti-X™ 293T	Takara Bio	Cat#632180
Human: hTERT-RPE1 (p53−/−, Rb−/−)	Titia de Lange, Rockefeller Univ.	N/A
Human: HeLa RMCE GFP-TRF1	Huaiying Zhang, Carnegie Mellon Univ.	N/A
		
**Experimental Models: Organisms/Strains**		
		
		
		
		
		
		
**Oligonucleotides**		
See Table S1		
siRNA targeting human TRF2 (#2 sequence from Takai et al. 2003): 5′-UGU GCU GGA GAU GAU UAA AAC-3′	IDT	N/A
siRNA targeting human TRF2 (#4 sequence from Takai et al. 2003): 5′-AUC GCU GGC GGA CCA UGA A-3′	IDT	N/A
siRNA targeting human TRF2 (sequence from Yang et al. 2015): 5′-CCA GAA GGA UCU GGU UCU UTT-3′	IDT	N/A
Scrambled RNAi (sequence from Yang et al. 2015): 5′-UUC UCC GAA CGU GUC ACG UTT-3′	IDT	N/A
*Silencer*™ Cy™3-labeled Negative Control No.1 siRNA	Thermo Fisher Scientific	Cat#AM4621
		
**Recombinant DNA**		
CDS: iLID	Bracha et al. 2018	N/A
CDS: TRF1 (NCBI Reference sequence: NM_003218.3)	IDT gBlock with codon optimization	N/A
CDS: TRF2 (NCBI Reference Sequence: NM_005652.5)	IDT gBlock with codon optimization	N/A
Plasmid: FM5-iLId-miRFP-TRF1	This paper	N/A
Plasmid: FM5-GFP-TRF2	This paper	N/A
Plasmid: FM5-miRFP-TRF1	This paper	N/A
Plasmid: FM5-miRFP-TRF2	This paper	N/A
Plasmid: FM5-sspB-mCherry	Sanders et al. 2020	N/A
Plasmid: FM5-sspB-mCherry-TRF1^A75P^	This paper	N/A
Plasmid: FM5-sspB-mCherry-TRF2	This paper	N/A
Plasmid: FM5-sspB-mCherry-TRF2^Acidic^	This paper	N/A
Plasmid: FM5-sspB-mCherry-TRF2^ΔB^	This paper	N/A
Plasmid: FM5-TRF1-mCherry-sspB	This paper	N/A
Plasmid: pCMV-dR8.91	Toettcher Lab, Princeton University	N/A
Plasmid: pMD2.G	Toettcher Lab, Princeton University	N/A
Plasmid: pHR-FUS_N_-mCherry-sspB	Bracha et al. 2018	N/A
Plasmid: pHR-FUS_N_-miRFP-TRF1	Shunsuke Shimobayashi (Brangwynne Lab, Princeton University)	N/A
Plasmid: pHR-NLS-iLID-EGFP-FTH1	Bracha et al. 2018	N/A
Plasmid: PSP	Sanders et al. 2020	N/A
Plasmid: VSVG	Sanders et al. 2020	N/A
Plasmid: EGFP-pBAD	Davidson Lab, Florida State University	Addgene Cat#54762
Plasmid: pet29b-SFP-His	Worthington & Burkart, 2006	Addgene Cat#75015
Plasmid: pET-H2A	Luger et al., 1999	N/A
Plasmid: pET-H2B	Luger et al., 1999	N/A
Plasmid: pET-H3	Luger et al., 1999	N/A
Plasmid: pET-H4	Luger et al., 1999	N/A
Plasmid: pGEM-3z/601	Lowary and Widom, 1998	Addgene Cat#26656
Plasmid: pOmnibac zz TEV YBBR TRF1	This paper	N/A
Plasmid: pOmnibac zz TEV YBBR TRF1^ΔA^	This paper	N/A
Plasmid: pOmnibac zz TEV YBBR TRF1^ΔHinge^	This paper	N/A
Plasmid: pOmnibac zz TEV YBBR TRF1^ΔIDR^	This paper	N/A
Plasmid: pOmnibac zz TEV YBBR TRF1^Hinge^	This paper	N/A
Plasmid: pOmnibac zz TEV YBBR TRF1^HingeMyb^	This paper	N/A
Plasmid: pOmnibac zz TEV YBBR TRF1^GSTHingeMyb^	This paper	N/A
Plasmid: pOmnibac zz TEV YBBR TRF1^Basic^	This paper	N/A
Plasmid: pOmnibac zz TEV YBBR TRF2	This paper	N/A
Plasmid: pOmnibac zz TEV YBBR TRF2^ΔB^	This paper	N/A
Plasmid: pOmnibac zz TEV YBBR TRF2^ΔHinge^	This paper	N/A
Plasmid: pOmnibac zz TEV YBBR TRF2^ΔIDR^	This paper	N/A
Plasmid: pOmnibac zz TEV YBBR TRF2^Hinge^	This paper	N/A
Plasmid: pOmnibac zz TEV YBBR TRF2^HingeMyb^	This paper	N/A
Plasmid: pOmnibac zz TEV YBBR TRF2^GSTHingeMyb^	This paper	N/A
Plasmid: pOmnibac zz TEV YBBR TRF2^GSTSub^	This paper	N/A
Plasmid: pOmnibac zz TEV YBBR TRF2 ^ΔTRFH^	This paper	N/A
Plasmid: pOmnibac zz TEV YBBR TRF2^Acidic^	This paper	N/A
Plasmid: pOmnibac zz TEV YBBR TRF1^A74D^	This paper	N/A
Plasmid: pOmnibac zz TEV YBBR TRF2^Y102F^	This paper	N/A
Plasmid: pOmnibac zz TEV YBBR POT1	This paper	N/A
Plasmid: pBig1a zz TEV YBBR TPP1 MBP TEV TIN2	This paper	N/A
Plasmid: pBig2ab zz TEV YBBR POT1 ZZ TEV TPP1 MBP TEV TIN2 ZZ TEV TRF1 (4comp1)	This paper	N/A
Plasmid: pBig1a zz TEV YBBR POT1 MBP TEV TPP1 MBP TEV TIN2 ZZ TEV TRF2 (4comp2)	This paper	N/A
Plasmid: pBig1a zz TEV YBBR RAP1 ZZ TEV TRF2	This paper	N/A
Plasmid: pLIB MBP TEV YBBR RAP1	This paper	N/A
Plasmid: pRST5-Spinach-39xTelG	This paper	N/A
		
**Software and Algorithms**		
Fiji (ImageJ 1.52p)	NIH	https://imagej.nih.gov/ij/
3D objects Counter (Fiji)	Bolte and Cordelières, 2006	
3D Multicoloc in 3D ImageJ suite (Fiji)	Ollion et al. 2013	
GraphPad PRISM 9.1.0	GraphPad	https://graphpad.com
MATLAB 2019b	MathWorks	https://www.mathworks.com/products/MATLAB.html
Python 3.7.10	Python Software Foundation	https://python.org
Origin 8.5.0 SR1	OriginLab Corporation	https://www.originlab.com/
		
**Other**		
IgG Sepharose beads	GE Healthcare	Cat#17096902
HisPur Ni-NTA beads	Thermo Fisher Scientific	Cat#88221
Amylose beads	New England BioLabs	Cat# E8021S
Superdex 200 Increase 10/300 GL	Cytiva	Cat#28-9909-44
Superdex 200 10/300 GL	Cytiva	Cat#17517501
NuPAGE 4-12% Bis-Tris gel	Thermo Fisher Scientific	Cat#NP0322BOX
PEG-Biotin cover slips	MicroSurfaces, Inc	Cat# Bio_02
Trans-Blot Turbo Mini 0.2 um PVDF transfer pack	Bio-Rad	Cat#1704156
40kDa Zeba spin desalting column	Thermo Fisher Scientific	Cat#87766
HiTrap SP HP	GE Life Sciences	Cat#95056-076
HiTrap DEAE-FF	Cytiva	Cat#17515401
Amersham Typhoon	GE Life Sciences	Cat#29238583
		
		
		
		
