# Supplementary material for: Compartmentalization of Telomeres through DNA-scaffolded Phase Separation
Source: Dev Cell. Author manuscript; Available in PMC 2022 Apr 24. (PMC8988007; doi:10.1016/j.devcel.2021.12.017)
Supplement: Supplement [file NIHMS1786045-supplement-Supplement.docx]

**Supplemental Tables**

| **Name** | **Sequence** |
| --- | --- |
| **2ds0ss** | 5’ GACCATGC TTAGGG TTATCATACAA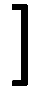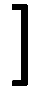  3’ CTGGTACG AATCCC **_2_** AATAGTATGTT |
| **3ds3ss** | 5’ GACCATGC TTAGGG TTATCATACAAGTTAGGGTTAGGGTTAGGG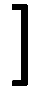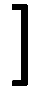  3’ CTGGTACG AATCCC **_3_** AATAGTATGTT |
| **8ds0ss** | 5’ GACCATGC TTAGGG TTATCATACAA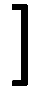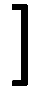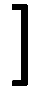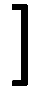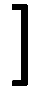  3’ CTGGTACG AATCCC **_8_** AATAGTATGTT |
| **8ds3ss** | 5’ GACCATGC TTAGGG TTATCATACAAGTTAGGGTTAGGGTTAGGG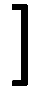  3’ CTGGTACG AATCCC **_8_** AATAGTATGTT |
| **13ds0ss** | 5’ GACCATGC TTAGGG TTATCATACAA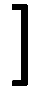  3’ CTGGTACG AATCCC **_13_** AATAGTATGTT |
| **39ds0ss** | 5’ GACCATGC TTAGGG TTATCATACAA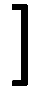  3’ CTGGTACG AATCCC **_39_** AATAGTATGTT |
| **Non-telomeric DNA** | 5’ CGTTCCGATGTCTTCAATCTGCAGGAGCTAGTCACGAACGAAGTACAAGGTTCTGTCCCAG  3’ GCAAGGCTACAGAAGTTAGACGTCCTCGATCAGTGCTTGCTTCATGTTCCAAGACAGGGTC  TGTAAGTAGCTTCACCAGGTAGATG  ACATTC |
| **Bio-8ds3ss** | 5’ GACCATGC TTAGGG TTATCATACAAGTTAGGGTTAGGGTTAGGG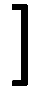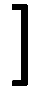  3’Bio-CTGGTACG AATCCC **_8_** AATAGTATGTT |
| **601-Widom** | 5’ GAGAATCCCGGTGCCGAGGCCGCTCAATTGGTCGTAGACAGCTCTAGCACCGCTTAAACG  3’ CTCTTAGGGCCACGGCTCCGGCGAGTTAACCAGCATCTGTCGAGATCGTGGCGAATTTGC  CACGTACGCGCTGTCCCCCGCGTTTTAACCGCCAAGGGGATTACTCCCTAGTCTCCAGGC  GTGCATGCGCGACAGGGGGCGCAAAATTGGCGGTTCCCCTAATGAGGGATCAGAGGTCCG  ACGTGTCAGATATATACATC  TGCACAGTCTATATATGTAG |
| **2x-Tel-Widom** | 5’ TCGAATTCTTAGGGTTAGGGTTACCCTGGAGAATCCCGGTGCCGAGGCCGCTCAATTGGT  3’ AGCTTAAGAATCCCAATCCCAATGGGACCTCTTAGGGCCACGGCTCCGGCGAGTTAACCA  CGTAGACAGCTCTAGCACCGCTTAAACGCACGTACGCGCTGTCCCCCGCGTTTTAACCGC  GCATCTGTCGAGATCGTGGCGAATTTGCGTGCATGCGCGACAGGGGGCGCAAAATTGGCG  GTTCCGGATTACTCCCTAGTCTCCAGGCACGTGTCAGATATATACATCCTGTGCTTAGGG  CAAGGCCTAATGAGGGATCAGAGGTCCGTGCACAGTCTATATATGTAGGACACGAATCCC  TTAGGGTTAGGATCCAG  AATCCCAATCCTAGGTC |
| **TERRA RNA** | 5’ UUAGGG **_10_**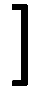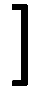 |

**Table S1: The list of telomeric and non-telomeric DNA and RNA constructs** **(bio: biotin); related to Figure 2 and STAR Methods.**

**Supplemental Figures**

**
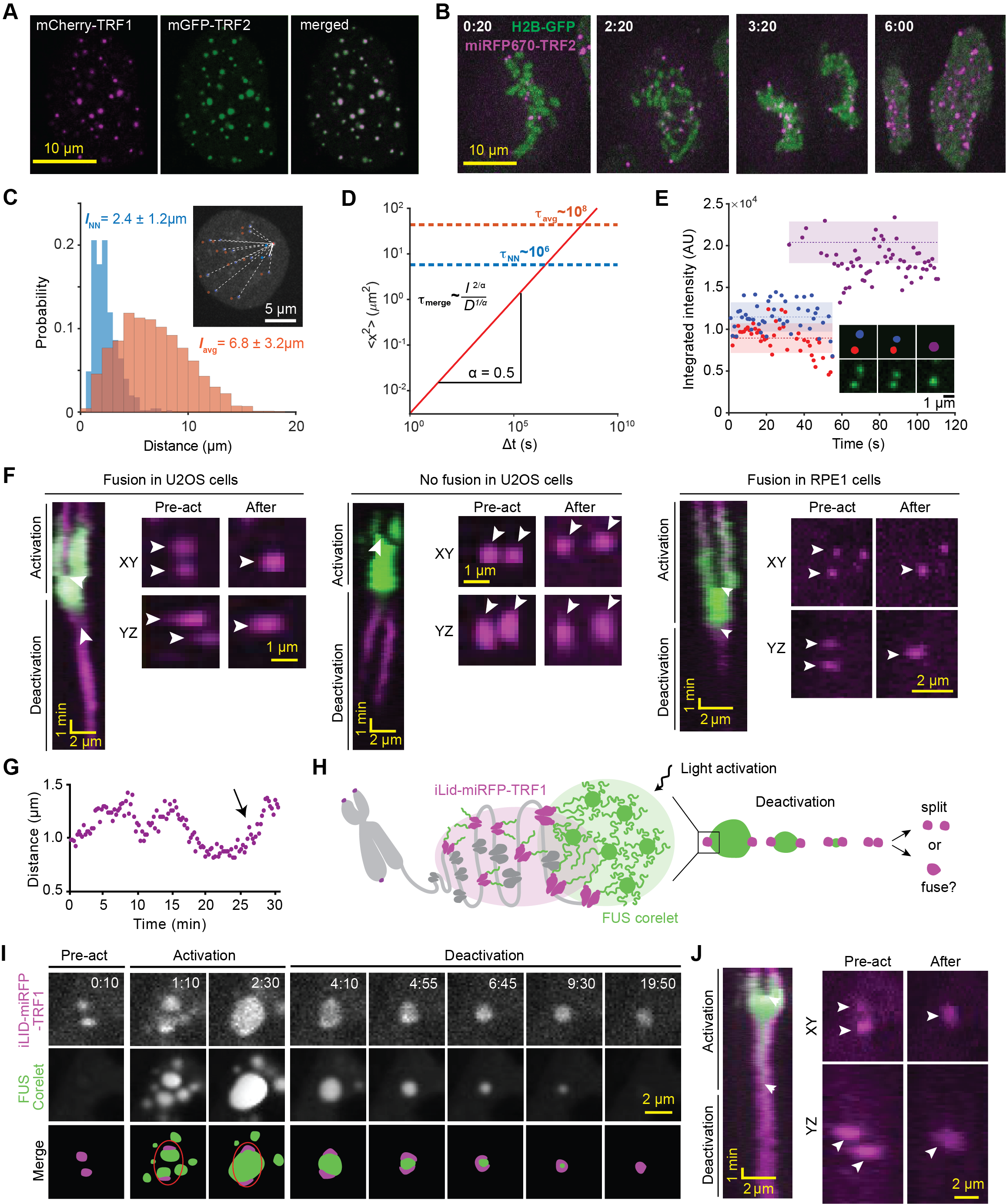
**

**­­­**

**Figure S1. Characterization of telomere behavior and TRF1 and TRF2 constructs in live cells; related to Figure 1. A.** U2OS cells expressing mCherry-TRF1 (left), mGFP-TRF2 (center), and a merged image showing co-localization of the two constructs (right). **B.** Panels from a 6-hour time-lapse movie, confirming miRFP-TRF2 localization at telomeres during metaphase. **C.** The histogram of the minimum pairwise distance (blue) and the average pairwise distances (orange) between telomeres. **D.** Linear extrapolation of the timescale predicted for telomeres to randomly contact their nearest neighbor (blue) and average other telomere (orange) using the MSD of TRF1. **E.** The integrated intensities of red and blue pseudo-colored telomeres add up as they coalesce into one purple telomere. **F.** Kymographs and before and after images of successful telomere mergers (left) or no merger event (middle) with FUS_N_-miRFP-TRF1 ‘seed’ construct in U2OS cells. The right panels show successful telomere coalescence of FUS_N_-miRFP-TRF1 ‘seed’ in hTERT-RPE1 cells, suggesting the fusion is not dependent on APBs, which are not present in this cell line. **G.** In cases of unsuccessful telomere coalescence, the telomeres relax back to their original distance away from each other. **H.** Unlike FUS_N_-miRFP-TRF1, iLID-miRFP-TRF1 becomes a seed upon light activation. TRF1 seed recruits FUS_N_ Corelet to telomeres. **I.** Pre-activation, activation, and deactivation of iLID-miRFP-TRF1 and FUS_N_ Corelet. The ellipse in merged images shows the local activation pattern. **J.** (Left) Kymograph shows that the two telomeres merge and remain as a single spot after deactivation. White arrowheads indicate the merging of droplets and telomeres. (Right) XY and YZ views of the telomeres before and after the coalescence event. White arrowheads mark two telomeres that coalesce upon activation.

**
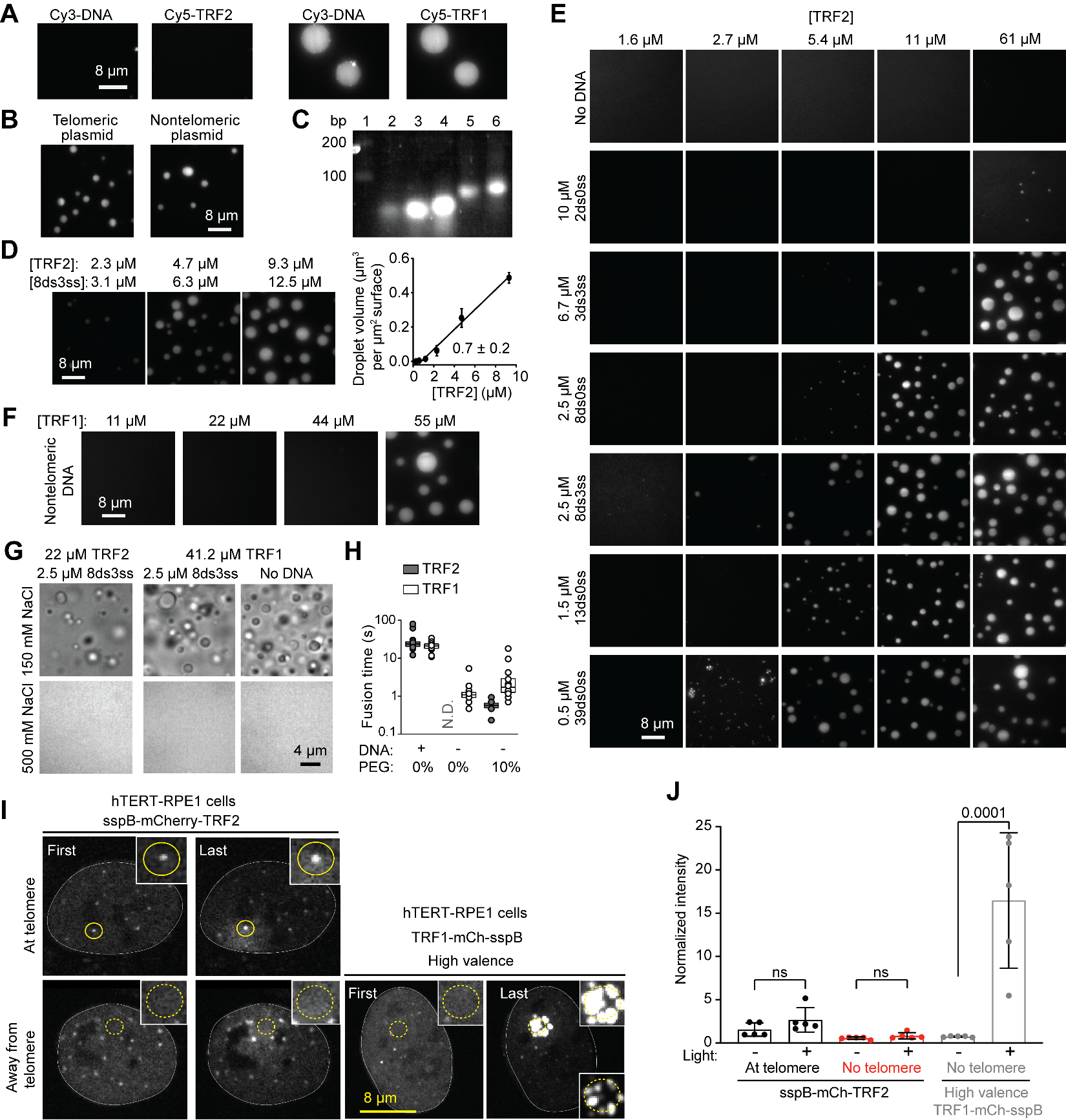
**

**Figure S2. Phase separation of TRF2 and TRF1 *in vitro*; related to Figure 2. A.** 10 µM TRF2 forms droplets with 270 ng/µL DNA plasmid containing either 39 telomeric repeats (pRST5 vector, modified) (left) or no telomeric repeats (pFastBac vector, Addgene 55221) (right). **B.** TRF2 does not stimulate phase separation with 2.5 µM of the same length of non-telomeric DNA (left), whereas non-telomeric DNA partitions into the TRF1 droplets (right). TRF1 and TRF2 concentrations were 51 and 61 µM; respectively. **C.** A 2% agarose gel stained with GelRed (1: Marker, 2: 8ds0ss sense oligo, 3: 8ds3ss sense oligo, 4: 8ds antisense oligo, 5: 8ds0ss after hybridization, and 6: 8ds3ss after hybridization). The double-stranded DNA substrates migrate to a higher molecular weight than the individual ssDNA oligos. 75 ng of DNA were used in each sample. **D.** (Left) Condensates were formed under a constant ratio of TRF2 and telomeric DNA. (Right) The total volume of the condensates settled per micron squared area on the coverslip (mean ± SD, n = 20 with two technical replicates). Linear fit (solid line) reveals *c*_sat_ (± SE). **E.** Example images of Cy3-TRF2 in the presence and absence of telomeric DNA show that increasing the valency of the telomeric DNA substrates stimulates LLPS of TRF2 *in vitro*. The concentration of dsTEL tracts was fixed to 20 µM while varying the number of telomeric repeats in the DNA substrates. **F.** Example images show the formation of TRF1 droplets with 2.5 µM non-telomeric DNA. **G.** Brightfield images show that TRF2 and TRF1 droplets are dissolved at 500 mM NaCl. **H.** Fusion times of TRF2 and TRF1 droplets in the presence and absence of telomeric DNA and crowding agent (N.D.: not determined). TRF2 concentration was set to 22 µM, TRF1 concentration was set to 44 µM, + DNA indicates 2.5 µM 8ds3ss, and + PEG indicates 10% PEG-8000. The center and edges of the box represent the median with the first and third quartiles. **I.** sspB-mCherry-TRF2 and TRF1-mCh-sspB were expressed with the Corelet system in hTERT-RPE1 cells and localize to telomeres before light activation. sspB-mCherry-TRF2 was locally activated at a single telomere (top) and away from telomeres (bottom); shown are the first and last frames of activation. Insets show activated region. At high valence, local activation of TRF1-mCh-sspB can form droplets even away from telomeres (right), bottom inset shows alternate contrast of top inset. **J.** Quantification of change in intensity upon local activation, at and away from existing telomeres for sspB-mCh-TRF2 and TRF1-mCh-sspB in hTERT-RPE1 cells (n = 5 cells analyzed for each condition). Intensities were normalized to average intensity at non-activated telomeres in the same cell. P-values were calculated by one-way ANOVA with multiple comparisons.

**
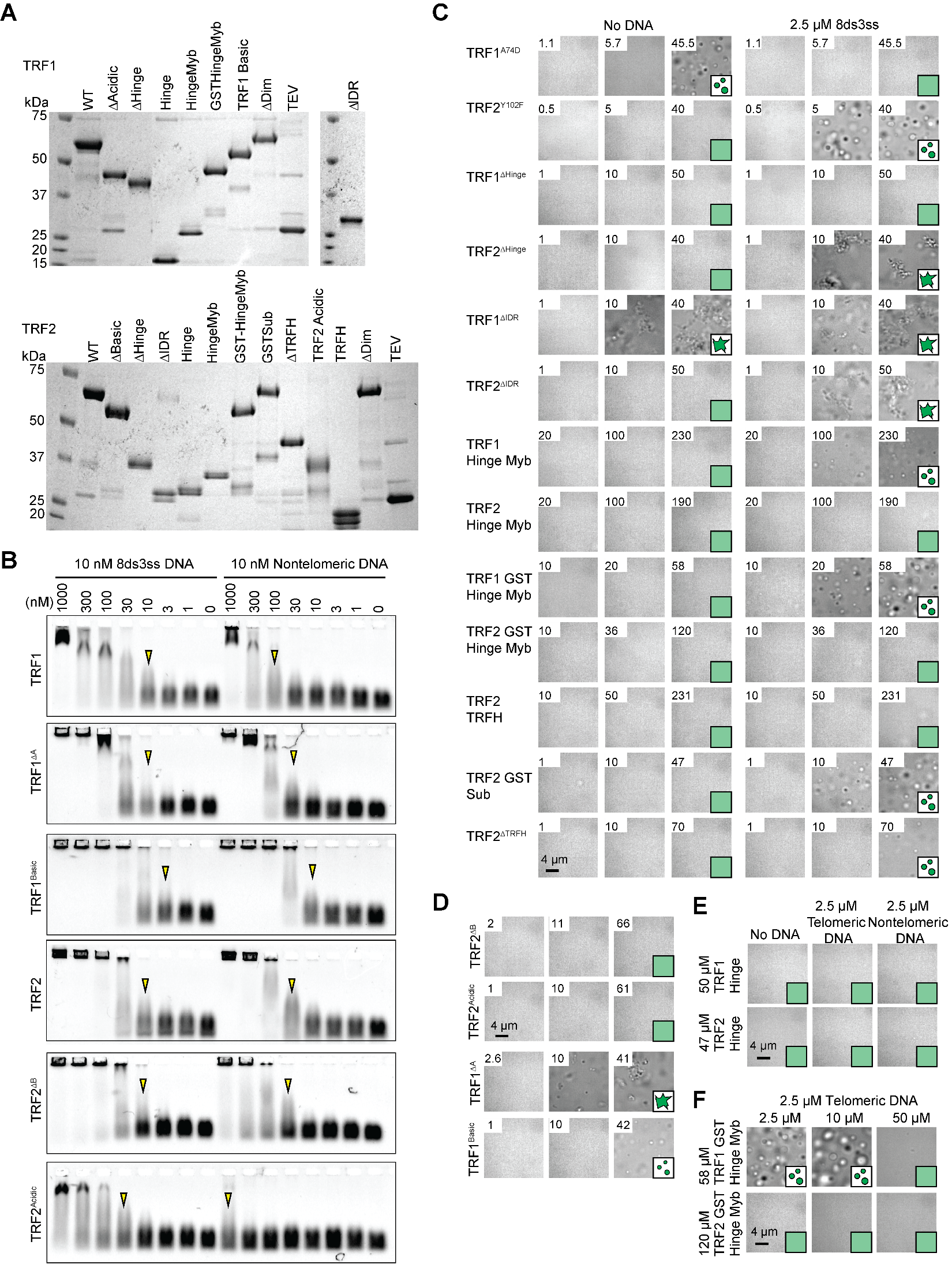
**

**Figure S3. Biochemical characterization and phase separation of the TRF1 and TRF2 mutants; related to Figures 2 and 3. A.** Denaturing gel pictures of purified TRF1 and TRF2 mutants. The gels were stained with Coomassie. **B.** EMSA gels using Cy3 labeled 10 nM telomeric or nontelomeric DNA and decreasing protein concentration. Arrows indicate the minimum protein concentration for each condition with a noticeable shift in the DNA band. **C.** Brightfield images of native and truncated TRF2 and TRF1 constructs in the presence and absence of 2.5 µM 8ds3ss. **D.** In the absence of DNA, TRF2^ΔB^, TRF2^Acidic^, and TRF1^ΔA^ do not form liquid droplets, whereas TRF1^Basic^ forms small droplets only at high protein concentrations. **E.** Brightfield images of the hinge domain of TRF2 and TRF1 without DNA, and with 2.5 µM telomeric (8ds3ss) or non-telomeric DNA. **F.** Hinge-Myb of TRF1 and TRF2 were homodimerized with an N-terminal glutathione serin transferase (GST) tag. An increase in DNA concentration inhibits phase separation of TRF1 GST-Hinge-Myb similar to native TRF1 but does not affect TRF2 GST-Hinge-Myb. In C-D, the protein concentration is shown in µM at the top left corner. In C-F, the condensate state at the highest protein concentration is shown in the bottom right corner.


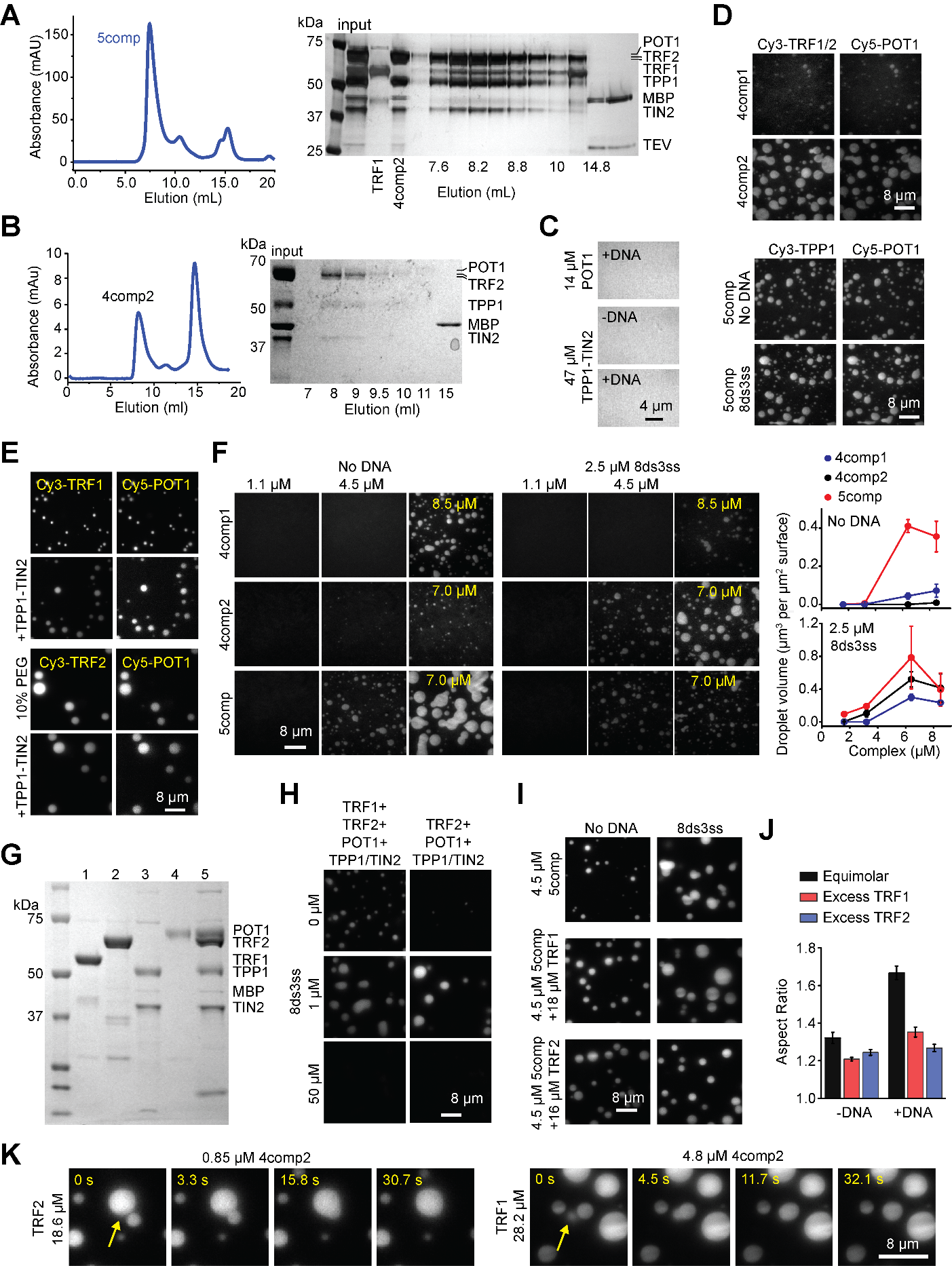


**Figure S4. Phase separation of shelterin is driven by TRF1 and TRF2; related to Figure 4.** **A.** UV absorbance (left) and silver-stained denaturing gel (right) show that 5comp elutes as a single complex from a gel filtration column. **B.** UV absorbance (left) and silver-stained denaturing gel (right) show that 4comp2 elutes as a single complex from a gel filtration column. **C.** POT1 and coexpressed TPP1-TIN2 do not form higher-order structures. **D.** (Left) Equimolar POT1 partitions into droplets formed by 8.5 µM 4comp1 or 7.0 µM 4comp2 in the presence of 2.5 µM 8ds3ss. (Right) Equimolar TPP1 and POT1 partition into droplets formed by 5.6 µM 5comp in the absence or presence of 2.5 µM 8ds3ss. **E.** 0.5 µM Cy5-POT1 partitions into droplets formed by 15.7 µM Cy3-TRF1 or 16.6 µM Cy3-TRF2 in the absence of DNA and with or without the addition of 7.8 µM coexpressed TPP1 and TIN2. 10% PEG-8000 was added to trigger droplet formation of TRF2 in the absence of DNA. **F.** (Left) 4comp1 and 5comp complexes phase separate without DNA, whereas 4comp2 shelterin phase separates most strongly with telomeric DNA. (Right) The total volume of shelterin condensates settled per micron squared area on the coverslip as a function of complex concentration (mean ± SD, n = 20 with two technical replicates). **G.** A denaturing gel of individual shelterin components as well as 4comp2 purified from insect cells (Lane 1: TRF1, 2: TRF2, 3: coexpressed TPP1 and TIN2, 4: POT1, 5: 4comp2). **H.** Mixing of 4.5 µM TRF2, TRF1, TPP1-TIN2, and POT1 separately results in phase separation with similar DNA dependence as the co-purified 4comp2 or 5comp complexes. **I.** While the equimolar 5comp complex forms nonspherical condensates in the presence of 8ds3ss, adding excess TRF1 or TRF2 results in the formation of spherical droplets. **J.** Aspect ratios of 5comp droplets in the presence and absence of excess 18.0 µM TRF1 or 16.4 µM TRF2 with 2.5 µM 8ds3ss (mean ± SE; from left to right, n = 648, 1383, 1389, 611, 832, and 1159 droplets). **K.** 4comp2 droplets formed with 2.5 µM 8ds3ss fuse within 30 s in the presence of excess TRF1 or TRF2.

**
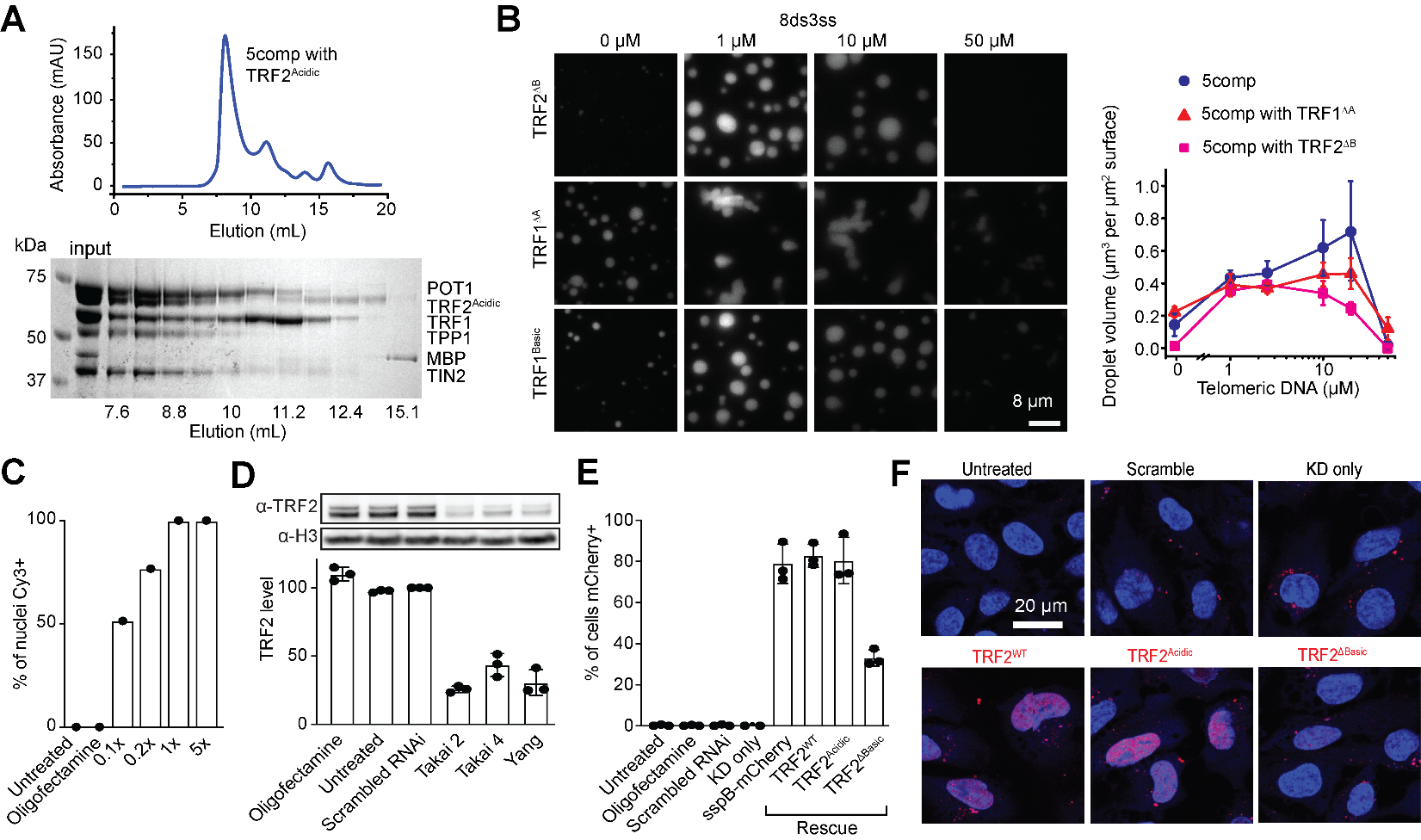
**

**Figure S5. TRF2 knockdown efficiency in cells and *in vitro* phase separation of shelterin complexes** **assembled with TRF1 and TRF2 mutants; related to Figure 5. A.** *In vitro* reconstitution of 5comp with TRF2^Acidic^ substituted for TRF2^WT^. UV absorbance (left) and denaturing gel (right) show that 5comp with TRF2^Acidic^ elutes primarily as a single complex from a gel filtration column. **B.** (Left) Replacing TRF1 with TRF1^ΔA^ or TRF1^Basic^ and replacing TRF2 with TRF2^ΔB^ in 5comp shelterin does not strongly affect DNA-dependent condensation of shelterin. The complex concentration was set to 4.5 µM. (Right) The total volume of condensates settled per micron squared area on the coverslip as a function of complex concentration (mean ±SD, n = 20 with two technical replicates). **C.** Percentage of nuclei expressing Cy3-labeled control siRNA to estimate siRNA transfection efficiency in hTERT-RPE1 cells. **D.** (Top) Western blot comparing endogenous and knockdown TRF2 levels (anti-TRF2) and loading control (anti-H3). (Bottom) Quantification of TRF2 level for each condition in 3 biological replicates. **E.** Percentage of cells expressing mCherry constructs, indicating infection efficiency. **F.** Example images show the mCherry signal (red) in hTERT-RPE1 cells under different conditions. Nuclei of cells are labeled with Hoechst (blue).

**
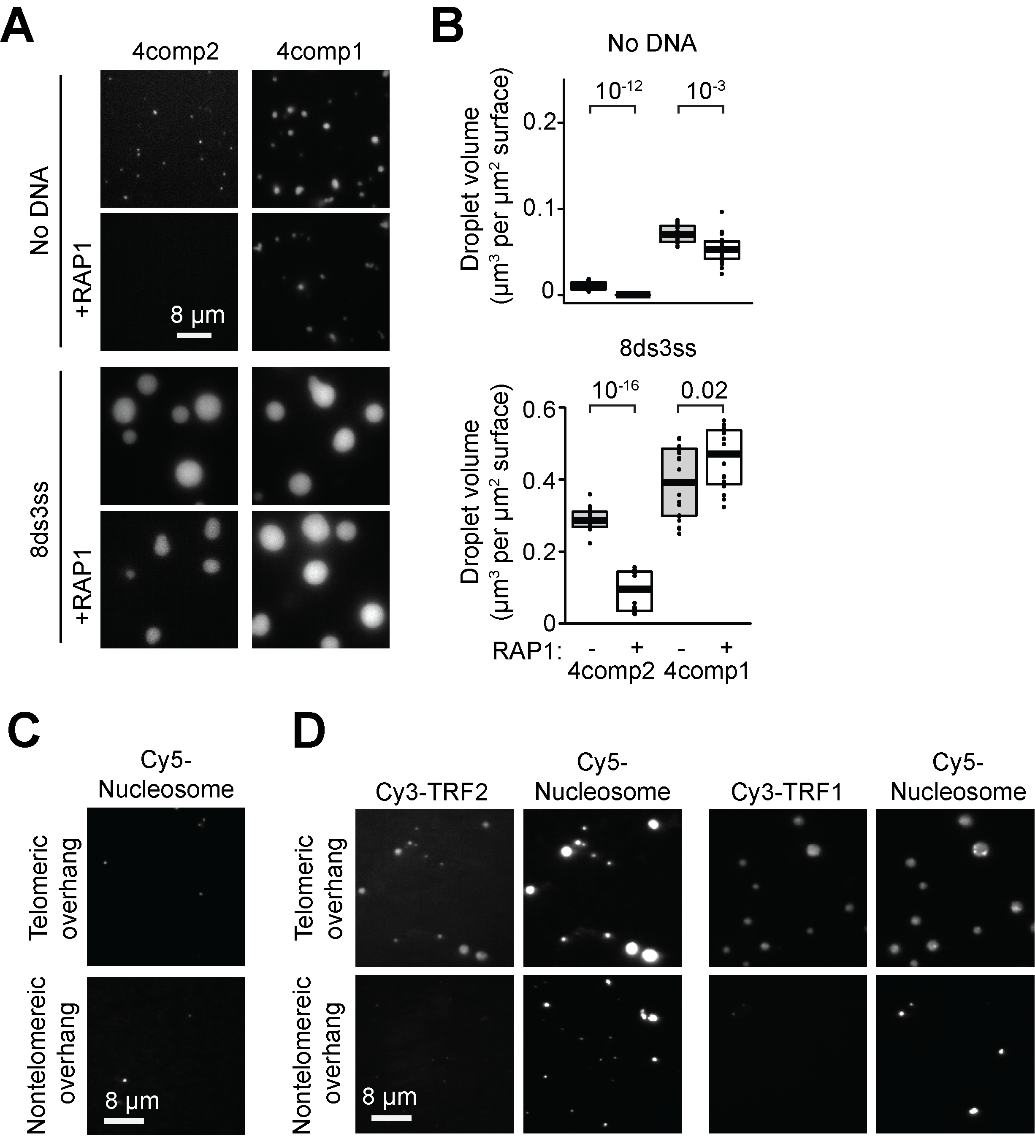
**

**Figure S6. Phase separation of shelterin in the presence of RAP1 and nucleosomes; related to Figure 6. A.** Shelterin droplets formed with or without equimolar RAP1 and in the presence or absence of 2.5 µM 8ds3ss DNA. Complex concentration was set at 4.5 µM for 4comp2 and 8.5 µM for 4comp1. **B.** The total volume of shelterin condensates settled per micron squared area on the coverslip in the absence or presence of RAP1. The center and edges of the box represent the median with the first and third quartile (n = 20 with two technical replicates). The p values were calculated from a two-tailed t-test. **C.** Without shelterin components, 0.5 µM mononucleosomes with telomeric or non-telomeric DNA overhangs do not phase separate in solution. **D.** 0.5 µM mononucleosomes with telomeric DNA overhangs (TTAGGGTTAGGG on each overhang) trigger droplet formation of TRF1 and TRF2 (at 42 and 37 µM, respectively), while 0.5 µM mononucleosomes wrapped with non-telomeric DNA do not promote phase separation of TRF2 or TRF1.


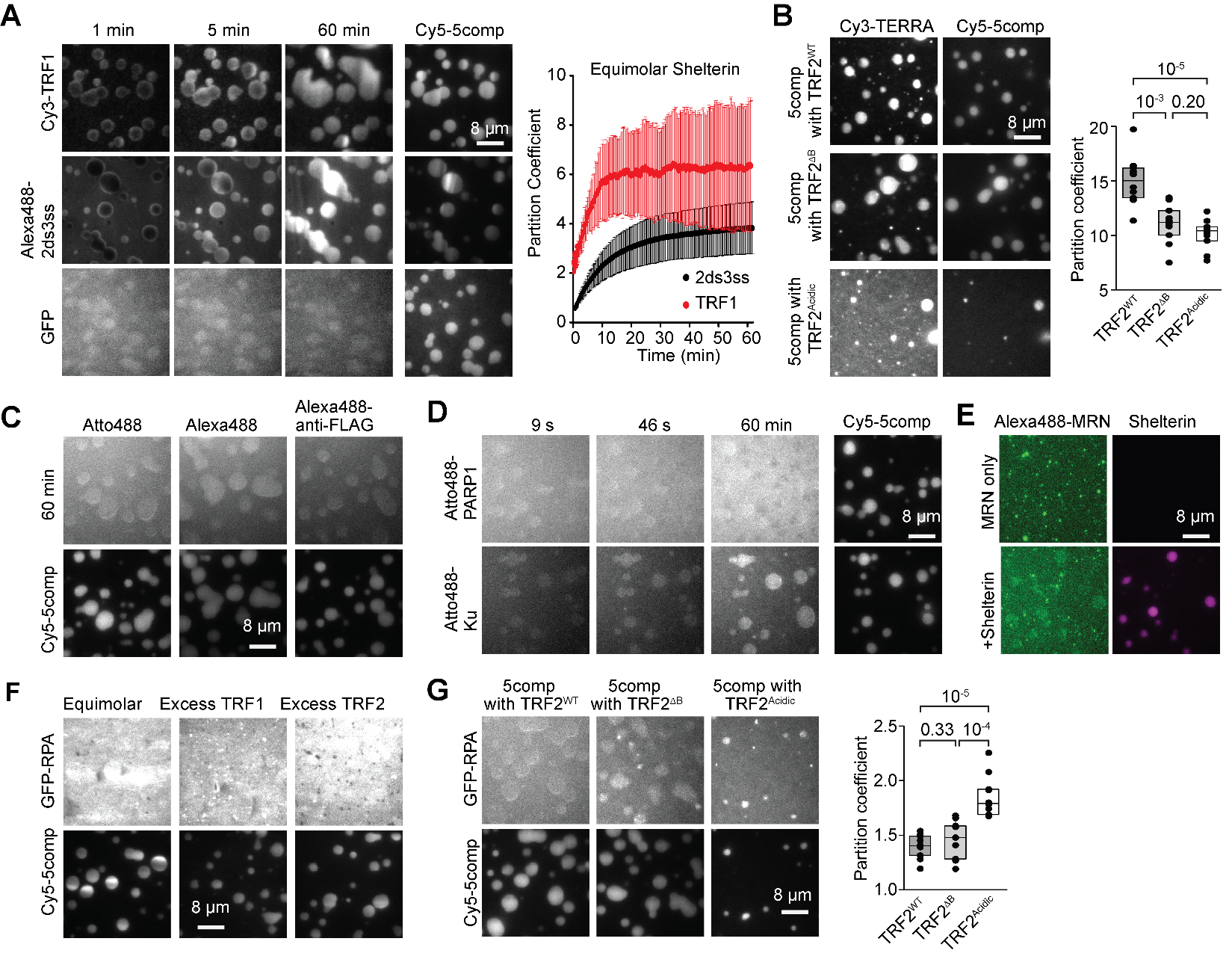


**Figure S7. Partitioning of telomeric and non-telomeric factors into shelterin droplets settled on telomeric DNA; related to Figure 7. A.** (Left) TRF1 (1.0 µM) and 2ds3ss DNA (2.5 µM) partition into 5comp shelterin droplets (7.6 µM) settled on surface-immobilized 8ds3ss DNA, while GFP (10.0 µM) is distributed uniformly over time. (Right) Partitioning of 2.5 µM 2ds3ss DNA or 1.0 µM TRF1 into equimolar 7.6 µM 5comp droplets settled on surface-immobilized 8ds3ss DNA over 1 h (mean ± SD, *n* = 3 droplets). **B.** (Left) 100 nM Cy3-TERRA is introduced to 5comp droplets assembled with Cy5-labeled TRF2^WT^ (7.6 µM complex; top), TRF2^ΔB^ (9.5 µM complex; middle), or TRF2^Acidic^ (9.5 µM complex; bottom) (mean ± SD, *n* = 3 droplets). (Right) The partition coefficient of TERRA in 5comp droplets after 60 min incubation. The center and edges of the box represent the median with the first and third quartile (n = 10 droplets per condition). The p-values were calculated from a two-tailed t-test. **C.** 100 nM Atto488 and 100 nM Alexa488 dye controls, and 100 nM Alexa488-anti-FLAG are distributed uniformly inside and outside of 7.6 µM 5comp shelterin droplets. **D.** 100 nM Atto488-PARP1 or Atto488-Ku are introduced to 7.6 µM 5comp droplets settled on surface-immobilized 8ds3ss DNA. After 60 minutes, PARP1 and Ku have partition coefficients of 1.2 and 1.8, respectively. **E.** 15 nM Alexa488-MRN forms small puncta on surface-immobilized 8ds3ss DNA in the absence (top) or presence (bottom) of 7.6 µM 5comp droplets. **F.** 100 nM GFP-RPA is introduced to Cy5-5comp droplets formed in the presence or absence of excess TRF1 or TRF2 (7.6 µM equimolar shelterin (Top); 3.8 µM shelterin with 13.1 µM excess TRF1 (Middle); and 5.0 µM shelterin with 16.7 µM excess TRF2 (Bottom)). GFP-RPA is uniformly distributed after 60 min incubation. **G.** (Left) 100 nM GFP-RPA is introduced to 5comp droplets assembled with Cy5-labeled TRF2^WT^ (7.6 µM complex; top), TRF2^ΔB^ (9.5 µM complex; middle), or TRF2^Acidic^ (9.5 µM complex; bottom) (mean ± SD, *n* = 3 droplets). (Right) The partition coefficient of RPA into 5comp droplets after 60 min incubation. The center and edges of the box represent the median with the first and third quartile (n = 10 droplets per condition). The p-values were calculated from a two-tailed t-test.
